# Expression of Cyclin E1 in hepatic stellate cells is critical for the induction and progression of liver fibrosis and hepatocellular carcinoma in mice

**DOI:** 10.1038/s41419-023-06077-4

**Published:** 2023-08-24

**Authors:** Julia Otto, Anna Verwaayen, Christian Penners, Jana Hundertmark, Cheng Lin, Carina Kallen, Daniela Paffen, Tobias Otto, Hilmar Berger, Frank Tacke, Ralf Weiskirchen, Yulia A. Nevzorova, Matthias Bartneck, Christian Trautwein, Roland Sonntag, Christian Liedtke

**Affiliations:** 1 Department of Medicine III, University Hospital RWTH Aachen, Aachen, Germany; 2 Charité - Universitätsmedizin Berlin, Department of Hepatology and Gastroenterology, Campus Virchow-Klinikum and Campus Charité Mitte, Berlin, Germany; 3 Institute of Molecular Pathobiochemistry, Experimental Gene Therapy and Clinical Chemistry (IFMPEGKC), University Hospital RWTH Aachen, Aachen, Germany; 4 Department of Immunology, Ophthalmology and ENT, Complutense University School of Medicine, Madrid, Spain; 5 DWI – Leibniz Institute for Interactive Materials, Aachen, Germany; 6 Institute of Technical and Macromolecular Chemistry, RWTH Aachen University, Aachen, Germany

**Keywords:** DNA synthesis, Liver cancer

## Abstract

Hepatocellular carcinoma (HCC) is one of the most severe malignancies with increasing incidence and limited treatment options. Typically, HCC develops during a multistep process involving chronic liver inflammation and liver fibrosis. The latter is characterized by the accumulation of extracellular matrix produced by Hepatic Stellate Cells (HSCs). This process involves cell cycle re-entry and proliferation of normally quiescent HSCs in an ordered sequence that is highly regulated by cyclins and associated cyclin-dependent kinases (CDKs) such as the Cyclin E1 (CCNE1)/CDK2 kinase complex. In the present study, we examined the role of Cyclin E1 (*Ccne1*) and *Cdk2* genes in HSCs for liver fibrogenesis and hepatocarcinogenesis. To this end, we generated conditional knockout mice lacking *Ccne1* or *Cdk2* specifically in HSCs (*Ccne1*^∆HSC^ or *Cdk2*^∆HSC^). *Ccne1*^∆HSC^ mice showed significantly reduced liver fibrosis formation and attenuated HSC activation in the carbon tetrachloride (CCl_4_) model. In a combined model of fibrosis-driven hepatocarcinogenesis, *Ccne1*^∆HSC^ mice revealed decreased HSC activation even after long-term observation and substantially reduced tumor load in the liver when compared to wild-type controls. Importantly, the deletion of *Cdk2* in HSCs also resulted in attenuated liver fibrosis after chronic CCl_4_ treatment. Single-cell RNA sequencing revealed that only a small fraction of HSCs expressed *Ccne1*/*Cdk2* at a distinct time point after CCl_4_ treatment. In summary, we provide evidence that *Ccne1* expression in a small population of HSCs is sufficient to trigger extensive liver fibrosis and hepatocarcinogenesis in a *Cdk2*-dependent manner. Thus, HSC-specific targeting of *Ccne1* or *Cdk2* in patients with liver fibrosis and high risk for HCC development could be therapeutically beneficial.

## Introduction

Hepatocellular carcinoma (HCC) is the fifth most common cancer worldwide, with a high mortality rate, increasing incidence, and limited treatment options. Development of HCC is complex and typically based on a multi-step process involving chronic hepatitis and hepatic fibrogenesis, eventually resulting in malignant transformation of hepatocytes [1]. A key event in liver fibrogenesis is the activation of hepatic stellate cells (HSCs). HSCs are defined as vitamin A-storing pericytes that constitute 5–8% of all liver cells [2]. HSC activation requires induction of pro-fibrotic genes such as *Acta2* (encoding alpha-smooth muscle actin), *Col1a1* (encoding collagen type I alpha 1), and *Pdgfrb* (encoding Platelet-derived growth factor receptor-beta) [3]. This eventually results in the transdifferentiation of resting HSC into collagen-producing, proliferating myofibroblasts [4]. This process also involves cell cycle re-entry and proliferation of the normally quiescent HSCs. Basically, the cell cycle is regulated by cyclins and associated cyclin-dependent kinases (Cdks). The transition from the quiescent state of the cell cycle into the phase of DNA synthesis is thought to be regulated by E-type cyclins Cyclin E1 (CCNE1) and Cyclin E2 (CCNE2) in complex with cyclin-dependent kinase 2 (CDK2) [5]. In this context, we previously demonstrated that *Ccne1* gene expression is induced in human and murine liver fibrosis [6]. Accordingly, constitutive, genetic knockout of *Ccne1*, but also systemic knockdown of *Ccne1* using small interfering RNA (siRNA) prevented the onset of liver fibrosis in mice in the established carbon tetrachloride (CCl_4_) model [6–8]. However, the major target cell population mediating the pro-fibrotic effect of *Ccne1* has not been clearly identified yet.

An abundance of studies has indicated that HSCs are also an important component of the HCC tumor microenvironment. Very recently, a comprehensive study using state-of-the-art single-cell sequencing technology in combination with cell-type specific genetic modifications revealed dual functions of HSCs in hepatocarcinogenesis, demonstrating that quiescent HSCs rather protect from HCC development, while activated HSCs promote HCC [9].

We have recently shown that *Ccne1* and *Cdk2* are essential for the initiation of HCC in mice [10]. While we were able to demonstrate that hepatocytes especially require *Cdk2* for hepatocarcinogenesis, the effector cells for the oncogenic effects of *Ccne1* in the liver have not yet been identified. Here, we tried to answer the question of whether the pro-fibrotic effect of *Ccne1* is triggered by a function in HSCs and whether this has an effect on downstream hepatocarcinogenesis. We provide evidence that the inactivation of *Ccne1* only in HSCs is sufficient to substantially reduce liver fibrosis but also HCC development in CCl_4_-mediated murine injury models, which is at least partially dependent on its canonical kinase subunit CDK2.

## Results

### Genetic ablation of *Ccne1*, specifically in HSCs, significantly reduces CCl_4_-mediated liver fibrosis

**Fig. 1:**
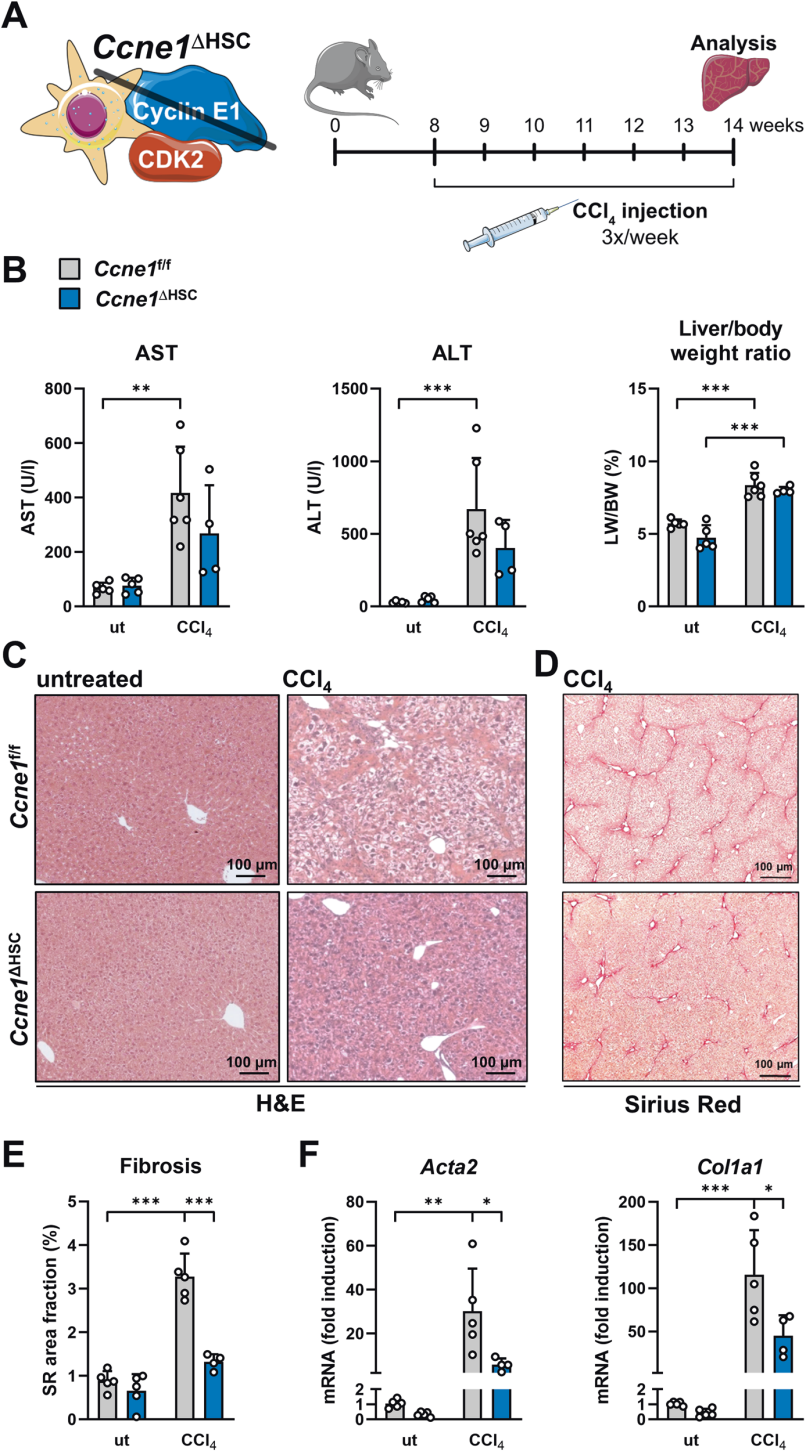
Genetic ablation of *Ccne1*, specifically in HSCs, reduces CCl_4_-mediated liver fibrosis. **A** Experimental setup: Mice with HSC-specific deletion of Cyclin E1 (*Ccne1*^ΔHSC^; *n* = 4) and control littermates (*Ccne1*^f/f^; *n* = 6) were injected with CCl_4_ three times a week, beginning with the age of 8 weeks. After 6 weeks (18 injections in total), mice were euthanized and examined for markers of liver damage and fibrosis progression. Control mice of both genotypes and matching ages were left untreated (ut). **B** Left**:** Determination of aspartate aminotransferase (AST) and alanine aminotransferase (ALT) activities. Values are given as units/liter (U/l). Right: Liver mass index was calculated as the liver weight (LW): body weight (BW) ratio in percent. **C** Representative Hematoxylin and Eosin (H&E) stained liver paraffin sections from *Ccne1*^f/f^ and *Ccne1*^ΔHSC^ mice after 6 weeks of CCl_4_-treatment. **D** Representative Sirius Red stainings from formalin-fixed paraffin-embedded liver sections. **E** Quantification of liver fibrosis from images in (**D**). Sirius Red-positive image areas were quantified using ImageJ software and calculated as a percentage of total tissue areas. **F** Determination of *Acta2* (encoding alpha-smooth-muscle actin, left) and *Col1a1* (encoding collagen type 1 alpha 1, right) mRNA expression by quantitative real-time PCR (qPCR). Expression values were normalized to the expression of the housekeeping gene *Gapdh* and calculated as fold induction in comparison to untreated *Ccne1*^f/f^ mice of the same genetic background and age. Data are expressed as means ± SD. *p ≤ 0.05; **p ≤ 0.005; ***p ≤ 0.001.

We generated mice with HSC-specific deletion of *Ccne1* (*Ccne1*^ΔHSC^) and challenged these animals and cre-negative littermates with repeated CCl_4_ injections for a period of 6 weeks (Fig. 1A). CCl_4_ treatment per se resulted in a significant increase in aspartate aminotransferase (AST) and alanine aminotransferase (ALT) activities and relative liver weights. However, we did not detect significant differences between *Ccne1*^f/f^ and *Ccne1*^ΔHSC^ mice regarding serum transaminase activities or relative liver mass under basal conditions or after CCl_4_ treatment (Fig. 1B). The livers of CCl_4_-treated *Ccne1*^f/f^ control littermates revealed extensive fibrosis as expected, which was significantly reduced in *Ccne1*^ΔHSC^ mice as evidenced by histological analysis (Fig. 1C), determination of collagen fiber formation (Fig. 1D, E) as well as lower induction of *Acta2* (encoding the HSC-activation marker alpha-smooth-muscle actin, i.e., αSMA) and down-regulation of *Col1a1* (encoding collagen type 1 alpha 1) (Fig. 1F). In summary, these data already indicate that *Ccne1* performs important pro-fibrotic functions in HSCs.

### *Ccne1* in HSCs drives hepatic fibrosis during HCC development

**Fig. 2:**
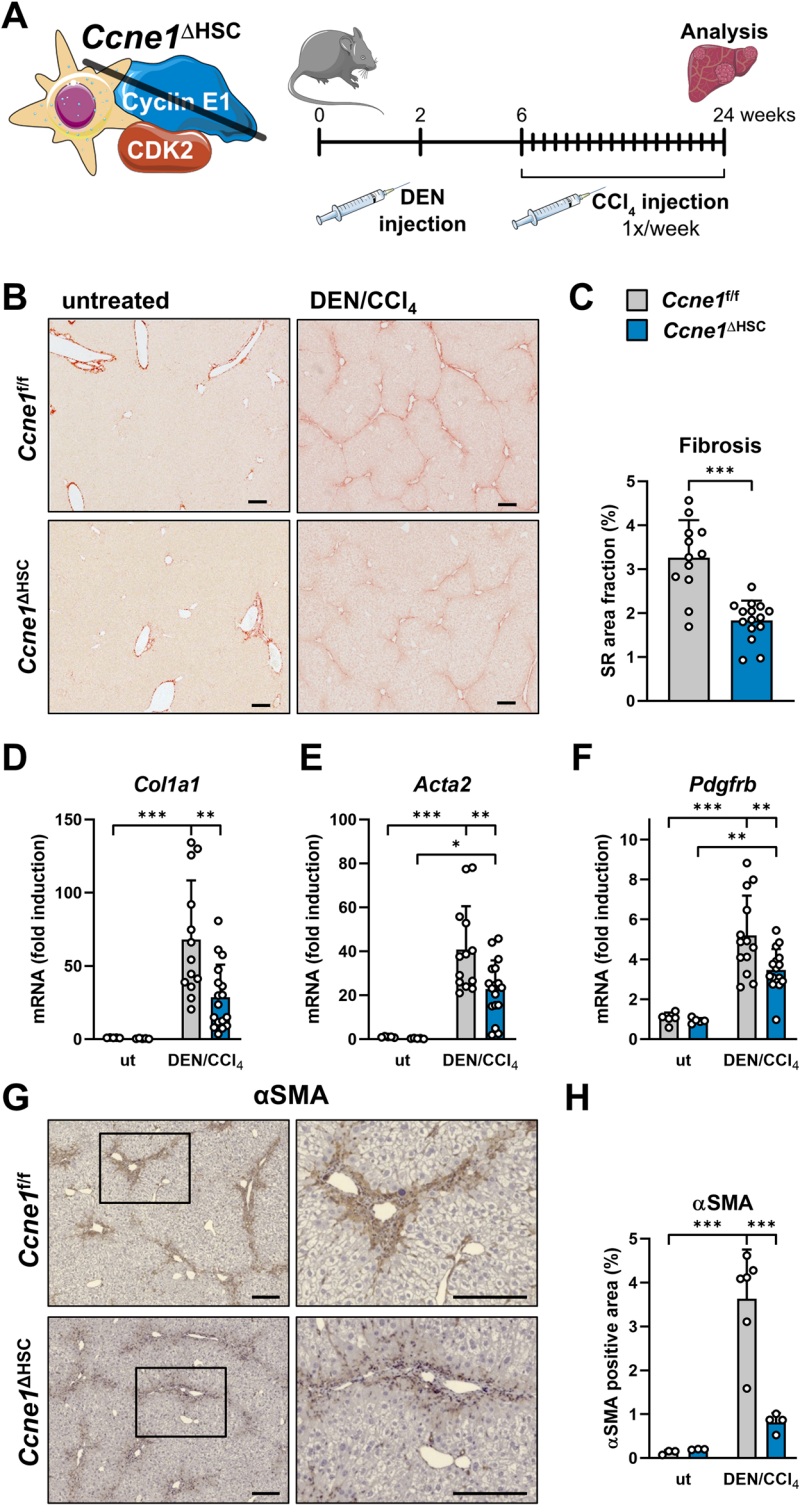
*Ccne1* in HSCs drives hepatic fibrosis during HCC development. **A** Experimental setup: *Ccne1*^ΔHSC^ mice (*n* = 15) and *Ccne1*^f/f^ littermates (*n* = 12) were subjected to the DEN/CCl_4_ HCC model. Mice were injected once with DEN on day 14 after birth to induce HCC, followed by weekly administration of CCl_4_ starting at the age of 6 weeks. Animals were euthanized at the age of 24 weeks for subsequent analysis. **B** Representative Sirius Red stainings from formalin-fixed paraffin-embedded liver sections. Scale bar: 100 µm. **C** Morphometric quantification of liver fibrosis. Sirius Red-stained images were analyzed for the percentage of stained image areas. **D**–**F** Gene expression analysis of (**D**). *Col1a1*, **E** *Acta2* and **F** *Pdfgfb* (encoding platelet-derived growth factor receptor beta) by qPCR. Gene expression values were normalized to *Gapdh* expression and calculated as fold induction in comparison to untreated (ut) *Ccne1*^f/f^ mice of the same genetic background and age. **G** Immunohistochemical staining for alpha-smooth-muscle actin (αSMA) of liver sections from DEN/CCl_4_ treated *Ccne1*^f/f^ and *Ccne1*^ΔHSC^ mice. Left: Overview; scale bar: 100 µm. Right: enlarged view of the marked area from left; scale bar: 100 µm. **H** Morphometric quantification of αSMA positive area from the IHC staining shown in (**E**) using ImageJ software with IHC toolbox. Data are expressed as mean ± SD. *p ≤ 0.05; **p ≤ 0.005; ***p ≤ 0.001.

Liver fibrosis is often considered a precursor to hepatocarcinogenesis [11]. In order to test whether the pro-fibrotic function of *Ccne1* in HSCs, as identified above, may also trigger the formation of liver cancer, we applied the *N*-nitrosodiethylamine (DEN)/CCl_4_ model [12]. This model combines the pro-inflammatory and pro-fibrotic effects of chronic CCl_4_ challenge with the hepatocarcinogenic effects of single DEN treatment and well reflects human pathogenesis from inflammatory liver injury via fibrosis to HCC. To this end, 2-week-old *Ccne1*^ΔHSC^ mice and *Ccne1*^f/f^ control littermates were injected once with DEN, followed by weekly injections of CCl_4_ from the 6^th^ to the 24th week of life (Fig. 2A). During this period, WT animals usually develop severe liver fibrosis, dysplastic lesions, and macroscopic HCCs.

As expected from previous publications, WT (i.e.*, Ccne1*^f/f^) mice developed pronounced liver fibrosis with excessive hepatic collagen deposition upon combined DEN/CCl_4_ treatment (Fig. 2B, C), which was accompanied by substantial induction of *Col1a1* gene expression and upregulation of *Acta2* (Fig. 2D, E). Importantly, the inactivation of *Ccne1*, specifically in HSCs, was sufficient to significantly reduce hepatic fiber formation, collagen expression, and HSC activation under the same conditions (Fig. 2B–E). In addition, deletion of *Ccne1* in HSCs led to significantly reduced expression of the *Pdgfrb* gene encoding platelet-derived growth factor receptor-beta (PDGFR-β, Fig. 2F), which is also typically expressed in activated HSCs [13]. Altogether, these data demonstrate that *Ccne1* is a pro-fibrotic key factor in HSCs which is essential for their activation and the onset of liver fibrogenesis in the setting of inflammatory hepatocarcinogenesis.

We analyzed the role of *Ccne1* for HSC activation in more detail by performing in situ stainings of αSMA in livers from DEN/CCl_4_-treated *Ccne1*^f/f^ and *Ccne1*^ΔHSC^ mice, thereby confirming significantly reduced αSMA protein expression and thus HSC activation in *Ccne1*^ΔHSC^ mice (Fig. 2G, H). Diminished αSMA protein expression through *Ccne1* depletion was especially evident in areas of tissue damage, where activated HSCs usually proliferate and produce an extracellular matrix (Fig. 2G, boxed areas). Of note, the inactivation of *Ccne1* in HSCs did not influence αSMA expression or collagen deposition in healthy mice under basal conditions (see Fig. 1E–G). In addition, the animals did not show a basal phenotype that could be attributed to the deletion of *Ccne1* in HSCs. Only the liver-to-body ratio showed a significant reduction in the untreated control animals (Supplementary Fig. 1A–G).

### HSC-specific deletion of *Ccne1* in mice leads to a reduced tumor burden during inflammatory hepatocarcinogenesis

**Fig. 3:**
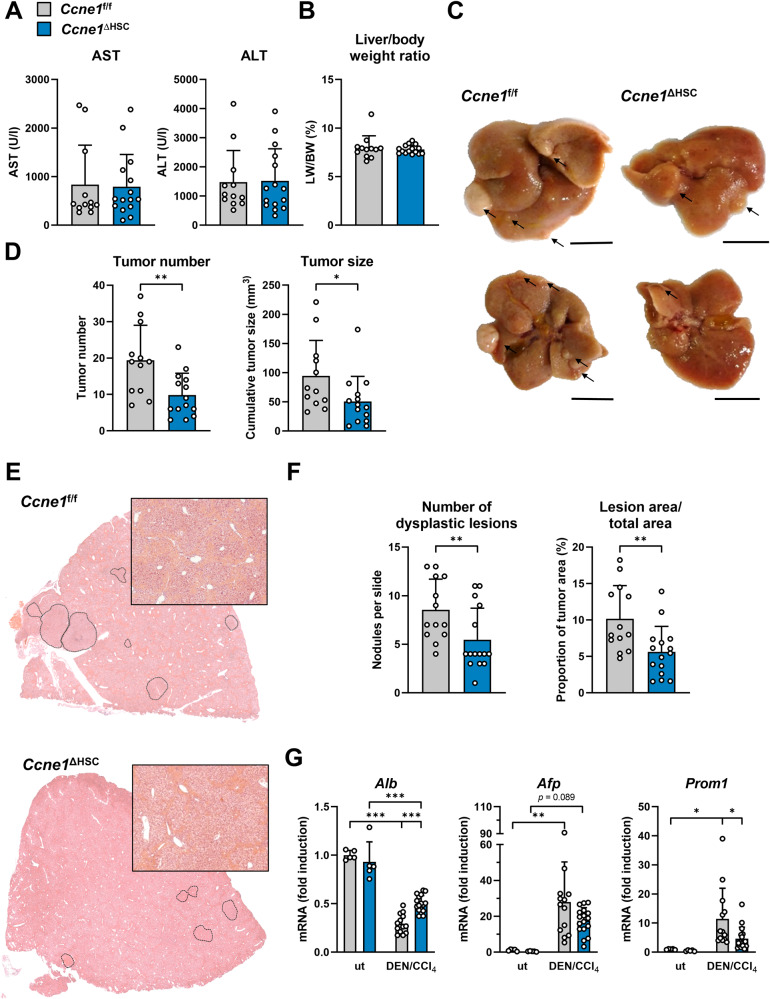
HSC-specific deletion of *Ccne1* in mice leads to a reduced number and size of dysplastic lesions during inflammatory hepatocarcinogenesis. *Ccne1*^ΔHSC^ mice (*n* = 15, blue bars) and *Ccne1*^f/f^ littermates (*n* = 12, gray bars) were subjected to the DEN/CCl_4_ HCC model as illustrated in Fig. 2A. Mice were sacrificed at the age of 24 weeks and analyzed for liver injury, liver histology, and HCC progression. **A** Determination of AST and ALT activities in Units/liter (U/l). **B** Determination of liver mass index calculated as the liver weight (LW): body weight (BW) ratio in percent. **C** Macroscopic appearance of explanted livers from *Ccne1*^f/f^ and *Ccne1*^ΔHSC^ mice. Representative liver tumors are highlighted by arrows. Scale bar: 1 cm. **D** Quantification of tumor burden in *Ccne1*^f/f^ and *Ccne1*^ΔHSC^ mice by macroscopic evaluation of HCC nodules. Left: Number of HCC nodules (tumor number); right: cumulative tumor diameter representing a measure of tumor size. **E** Representative H&E stained liver paraffin sections. Dysplastic liver areas are encircled. **F** Microscopic evaluation of histologic dysplastic areas displayed in (**E**). Left: The total number of dysplastic lesions per slide for each mouse is shown. Right: The proportion of dysplastic lesion areas was calculated as the lesion area/total liver area ratio in percent. **G** Determination of *Albumin* (*Alb*), *alpha-fetoprotein* (*Afp*), and *Prominin1* (*Prom1*, encoding CD133) mRNA expression by qPCR. Expression values were normalized to the expression of *Gapdh* and calculated as fold induction in comparison to untreated (ut) *Ccne1*^f/f^ mice. Data are expressed as mean ± SD. *p ≤ 0.05; **p ≤ 0.005; ***p ≤ 0.001.

We next investigated the impact of HSC-specific *Ccne1* deletion for hepatocarcinogenesis in the DEN/CCl_4_ model. *Ccne1*^ΔHSC^ mice did not reveal significant changes in AST or ALT activities (Fig. 3A) or in the relative liver-to-body weight ratio (Fig. 3B) after DEN/CCl_4_ treatment. However, the deletion of *Ccne1* in HSCs resulted in a significant reduction of absolute tumor numbers and the cumulative tumor size (Fig. 3C, D). In good agreement, the detailed histological analysis of tumorous changes revealed fewer dysplastic areas in *Ccne1*^ΔHSC^ mice compared to control animals (Fig. 3E, F). Interestingly, the fibrosis severity significantly correlated with the tumor number if all mice from both experimental groups were considered (Supplementary Fig. 2A). However, a more detailed analysis differentiated according to genotype showed that this correlation is only true for *Ccne1*^ΔHSC^ mice, but not for *Ccne1*^f/f^ controls (Supplementary Fig. 2B). This further indicates that specific, *Ccne1*-driven pro-fibrotic signals also trigger hepatocarcinogenesis.

We next examined if the absence of *Ccne1* in HSCs may affect the expression of genes encoding albumin (*Alb*), α-fetoprotein (*Afp*), and CD133 (*Prom1*), which are established markers for hepatocytes (*Alb*), de-differentiated hepatoma cells (*Afp*) or liver cancer stem cells (*Prom1*) [14–16]. As expected, DEN/CCl_4_ treatment resulted in reduced *Alb* gene expression in *Ccne1*^f/f^ and *Ccne1*^ΔHSC^ mice in comparison to untreated controls indicating HCC formation and thus de-differentiation of hepatocytes (Fig. 3G). However, this reduction of *Alb* was significantly lower in *Ccne1*^ΔHSC^ mice, which is in good agreement with the observed low HCC incidence in these mice. In contrast, *Afp* was up-regulated to the same extent in both *Ccne1*^f/f^ and *Ccne1*^ΔHSC^ mice, while deletion of *Ccne1* in HSCs resulted in significantly reduced induction of *Prom1* upon DEN/CCl_4_ treatment (Fig. 3G).

Altogether, our data suggest that the pro-fibrotic function of *Ccne1* in HSCs strongly contributes to the process of hepatocarcinogenesis.

### Deletion of *Ccne1*, specifically in HSCs, reduces proliferation and inflammation in the liver and modulates the myeloid tumor microenvironment

**Fig. 4:**
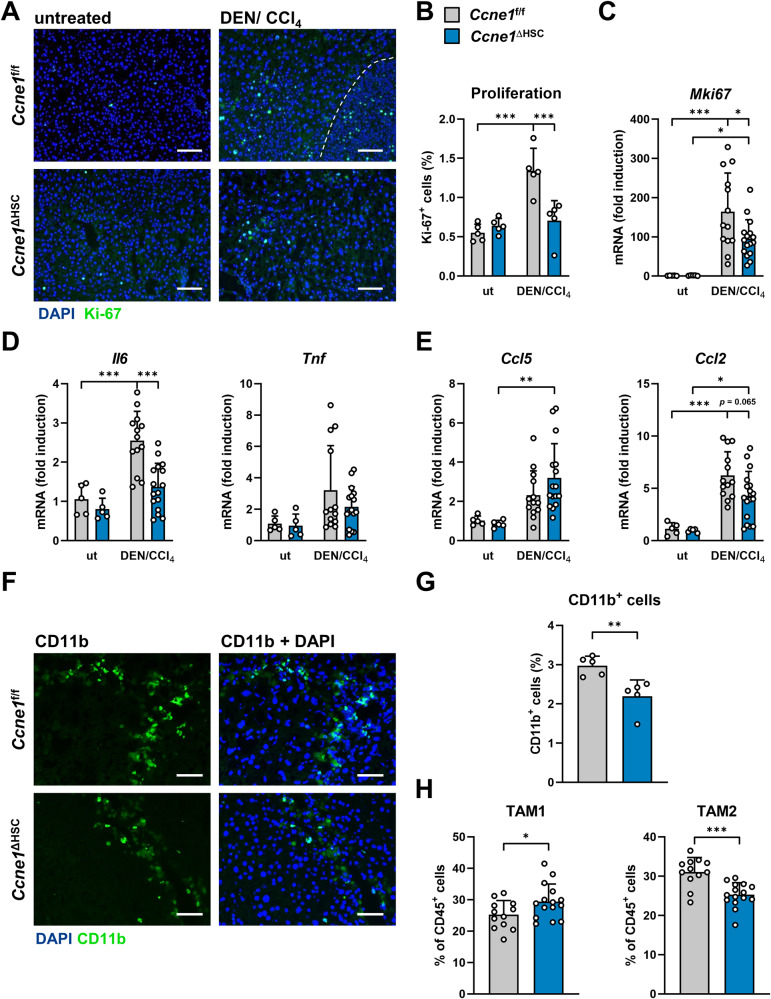
Deletion of Cyclin E1, specifically in HSCs, reduces proliferation and inflammation in the liver and modulates the myeloid tumor microenvironment. *Ccne1*^ΔHSC^ mice (*n* = 15, blue bars) and *Ccne1*^f/f^ littermates (*n* = 12, gray bars) were subjected to the DEN/CCl_4_ HCC model as illustrated in Fig. 2A. Mice were sacrificed at the age of 24 weeks. Explanted livers were analyzed for markers of proliferation and inflammation. **A** Representative immunofluorescence stainings for Ki-67 (green) on liver cryosections. Total nuclei are stained with DAPI (blue). The dashed line indicates the border of one representative dysplastic nodule. Scale bar: 100 µm. **B** Quantification of proliferating liver cells shown in (**A**) in percent. ut: untreated mice. **C**–**E** Gene expression analyses by qPCR. Expression values were normalized to the expression of *Gapdh* and calculated as fold induction in comparison to untreated (ut) *Ccne1*^f/f^ mice. **C** *Mki67* gene expression. **D** Gene expression of interleukin 6 (*Il6*) and tumor necrosis factor-α (*Tnf*). **E** Gene expression of chemokine (C–C motif) ligand 5 (*Ccl5*) and *Ccl2*. **F** Immunofluorescence staining for CD11b (green); total nuclei are stained with DAPI (blue). Scale bar: 100 µm. **G** Quantification of CD11b positive cells from the images shown in (**F**). Values are given as a percentage of total cells. **H** Immune cells were isolated from explanted livers, stained with fluorochrome-labeled antibodies, and then analyzed *via* flow cytometry (FACS). Percentage of TAM1 (defined as CD11b^+^, GR1^−^, MHCII^+^) and TAM2 (defined as CD11b^+^, GR1^+^, MHCII^−^) cells of CD45^+^ cells are depicted. Data are expressed as mean ± SD. *p ≤ 0.05; **p ≤ 0.005; ***p ≤ 0.001.

*Ccne1* is mostly known as a cell cycle regulator controlling the G_1_/S-phase transition [5]. As liver injury generated by the DEN/CCl_4_ model primarily induces compensatory proliferation and secondary liver cancer only, we next aimed to investigate whether the anti-fibrotic and anti-carcinogenic effects seen in DEN/CCl_4_-treated *Ccne1*^ΔHSC^ mice were also linked to an aberrant proliferation profile in the liver. Following DEN/CCl_4_ treatment, *Ccne1*^ΔHSC^ mice revealed a significantly reduced hepatic proliferation compared to controls as determined by Ki-67 protein- and corresponding *Mki67* gene expression (Fig. 4A–C). We further characterized the proliferating cell compartments in DEN/CCl_4_ treated *Ccne1*^f/f^ mice by co-staining of liver sections with Ki-67 and cell type-specific antibodies for hepatocytes (HNF4α), activated HSCs (αSMA) and immune cells (CD45), respectively. As expected, the majority of proliferating cells were identified as hepatocytes, while we detected rather few proliferating activated HSCs and almost no leukocytes with cell cycle activity (Supplementary Fig. 3A, B).

Regarding the pro-inflammatory response upon DEN/CCl_4_ challenge, we found a significant up-regulation of interleukin 6 (*Il6*) mRNA expression in fibrosis- and HCC-bearing *Ccne1*^f/f^ mice, which was reduced to almost background levels of untreated controls after HSC-specific deletion of *Ccne1* in *Ccne1*^ΔHSC^ mice. In contrast, the expression of the tumor necrosis factor-α gene (*Tnf*) was not significantly different between *Ccne1*^f/f^ and *Ccne1*^ΔHSC^ mice under both conditions (Fig. 4D). Thus, *Ccne1* seems to be involved in the control of *Il6* expression in the fibrotic and tumorous liver independent of *Tnf*.

HSCs can secrete the CC chemokine ligands 2 (CCL2) and 5 (CCL5) in response to pro-fibrotic signals, thereby inducing an overall chemotactic activity for monocytes (CCL2) and lymphocytes (CCL5), respectively [17]. In good agreement, *Ccl2* and *Ccl5* gene expression was induced in *Ccne1*^f/f^ mice after DEN/CCl_4_ treatment (Fig. 4E). Of note, inactivation of *Ccne1* in HSCs was associated with a tendency towards reduced mRNA expression of *Ccl2* (*p* = 0.065) compared to controls indicating that *Ccne1* could be directly or indirectly involved in immune cell recruitment.

In order to study this in more detail, we examined the myeloid cell fractions in the DEN/CCl_4_ treated liver of *Ccne1*^f/f^ and *Ccne1*^ΔHSC^ mice by immunofluorescence stainings and FACS analysis. Overall, we found a significantly decreased number of CD11b^+^ cells in mice with HSC-specific *Ccne1* deletion compared to control littermates (Fig. 4F, G). Moreover, FACS analysis revealed an altered polarization pattern of the mature myeloid (i.e., CD45^+^/CD11b^+^/Gr1^-^) compartment, referred to as tumor-associated macrophages (TAM). In detail, *Ccne1*^ΔHSC^ livers contained elevated numbers of MHCII-expressing TAMs (TAM1, Fig. 4H), which are considered pro-inflammatory or immune-competent [18]. Concomitantly, we detected a reduced number of MHCII-negative TAMs (TAM2, Fig. 4H) in *Ccne1*^ΔHSC^ HCC livers, which have been described as anti-inflammatory or immune-suppressive before [18]. This signature of reduced CCL2 signaling, myeloid cell infiltration, and TAM2-polarization has previously been associated with a predominantly anti-tumor microenvironment [17], resulting in an attenuated HCC burden [19]. Moreover, analysis of the lymphoid immune cell compartment by FACS did not show any differences between both groups (Supplementary Fig. 4). Altogether, our data indicate that *Ccne1* expression in HSCs affects the myeloid composition of the tumor environment during hepatocarcinogenesis.

### Deletion of *Cdk2*, specifically in HSCs, reduces CCl_4_-induced hepatic fibrogenesis

**Fig. 5:**
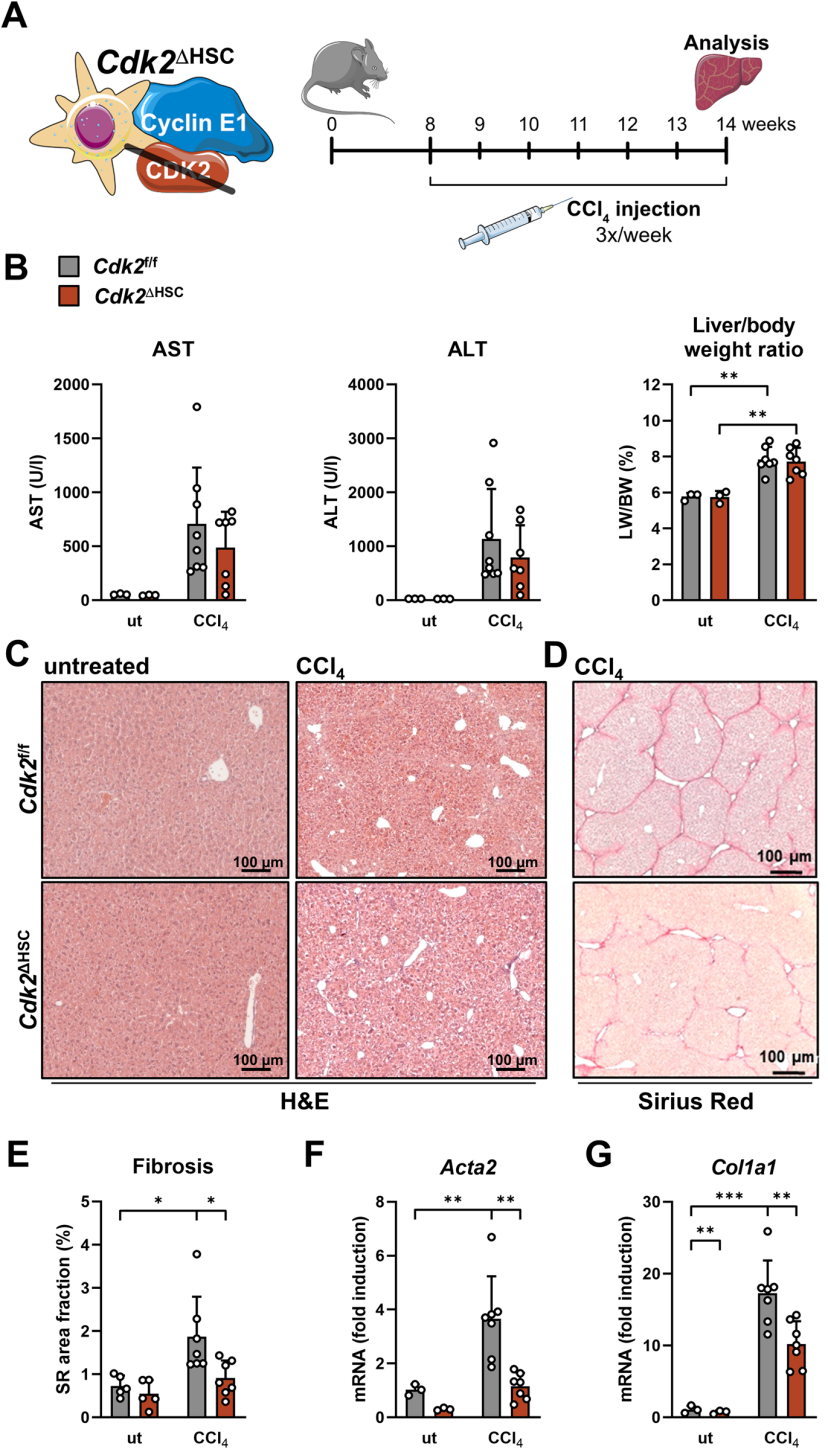
Deletion of *Cdk2* specifically in HSCs reduces CCl_4_-induced hepatic fibrogenesis. **A** Experimental setup: *Cdk2*^ΔHSC^ (*n* = 8, red bars) mice and *Cdk2*^f/f^ littermates (*n* = 7, gray bars) at the age of 8 weeks were challenged with CCl_4_ three times a week for 6 weeks to induce liver fibrosis. Mice were euthanized 48 h after the last injection and analyzed for liver histology, injury, and fibrosis progression. **B** Determination of AST and ALT activities in Units/liter (U/l). The liver mass index was calculated as the liver weight (LW): body weight (BW) ratio in percent. **C** Representative H&E-stained liver paraffin sections from *Cdk2*^f/f^ and *Cdk2*^ΔHSC^ mice after 6 weeks of CCl_4_-treatment. **D** Representative Sirius Red stainings of liver sections. **E** Morphometric quantification of liver fibrosis. Sirius Red stained images were analyzed for Sirius Red-positive image areas (percentage of total tissue areas) using ImageJ software. **F**, **G** Gene expression analysis of **F** *Acta2* and **G** *Col1a1* by qPCR. Expression values were normalized to the expression of *Gapdh* and calculated as fold induction in comparison to untreated *Cdk2*^f/f^ mice. ut: untreated. Data are expressed as mean ± SD. *p ≤ 0.05; **p ≤ 0.005; ***p ≤ 0.001.

E-type cyclins are considered regulatory subunits of CDK2. However, recent findings also point to several CDK2-independent functions of CCNE1, for instance, in the context of hepatocarcinogenesis [10, 20]. To clarify whether the identified pro-fibrotic role of CCNE1 depends on its canonical kinase subunit CDK2, we also subjected mice with HSC-specific deletion of *Cdk2* (*Cdk2*^ΔHSC^ mice) to the CCl_4_ fibrosis model for a total of 6 weeks (Fig. 5A). Liver transaminase activities (i.e., AST and ALT) and body mass indices were not different between WT control (i.e., *Cdk2*^f/f^) and *Cdk2*^ΔHSC^ mice under basal or treatment conditions, respectively (Fig. 5B). *Cdk2*^ΔHSC^ mice revealed improved liver architecture compared to control animals after CCl_4_ treatment (Fig. 5C). Importantly, *Cdk2*^ΔHSC^ mice showed a significant reduction of hepatic fibrosis compared to WT controls as determined by Sirius Red staining of fibers (Fig. 5D, E) and measurement of HSC activation markers *Acta2* and *Col1a1* (Fig. 5F, G). Thus, *Ccne1* and *Cdk2* both mediate pro-fibrotic properties in HSCs. Therefore, these data strongly indicate that the pro-fibrotic function of CCNE1 depends on functional CDK2.

### *Ccne1* drives pro-fibrotic and pro-carcinogenic properties of HSCs through *Cdk2*-dependent and *Cdk*2-independent mechanisms

**Fig. 6:**
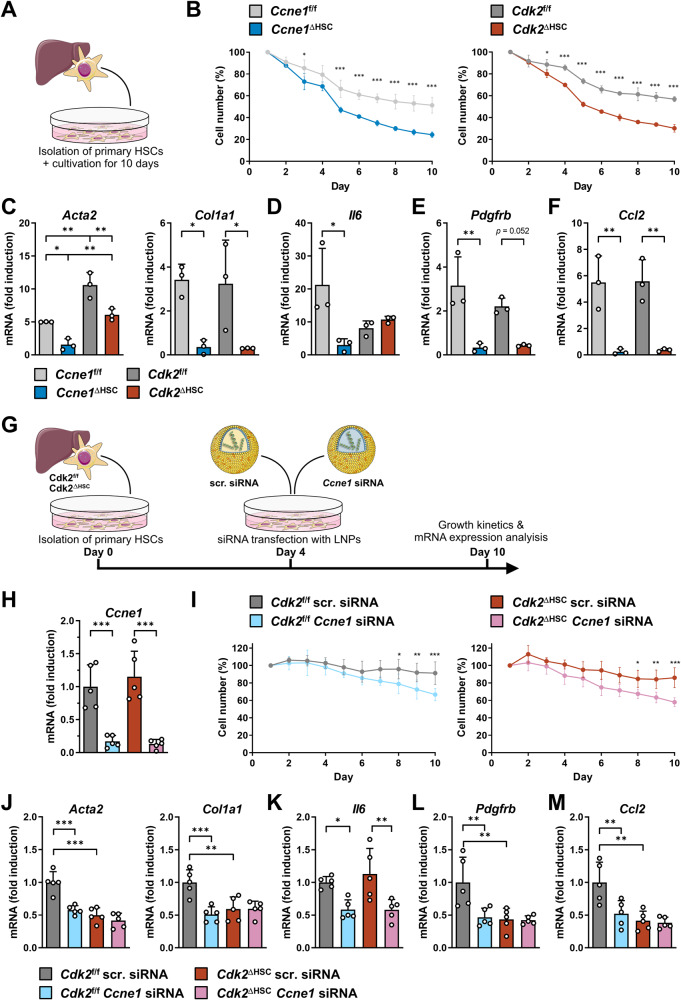
*Ccne1* drives pro-fibrotic and pro-carcinogenic properties of HSCs through *Cdk2*-dependent and *Cdk2*-independent mechanisms. **A** Experimental setup. Primary HSCs were isolated from *Ccne1*^ΔHSC^ and *Cdk2*^ΔHSC^ mice as well as from respective cre-negative (i.e., *Ccne1*^f/f^, *Cdk2*^f/f^) littermate controls. Cells were cultivated for up to 10 days (D0–D10) and analyzed at daily intervals. **B** Growth analysis of HSCs derived from *Ccne1*^ΔHSC^/*Ccne1*^f/f^ (left panel) and *Cdk2*^ΔHSC^/*Cdk2*^f/f^ mice (right panel). The same section of each culture plate was imaged daily and subjected to cell counting. Relative cell numbers were calculated as percent in relation to the cell number at day 1 of each biological replicate. **C**–**F**. Gene expression analysis of **C** *Acta2* (left), *Col1a1* (right), **D** *Il6*, **E** *Pdgfrb*, and **F** *Ccl2*. **G-M**. Concomitant inactivation of *Ccne1* and *Cdk2* in primary HSCs. **G**. Experimental setup. Primary HSCs were isolated from *Cdk2*^f/f^ and *Cdk2*^ΔHSC^ mice and cultivated as before. Four days after isolation, HSCs were transfected with lipid nanoparticles (LNPs) loaded with either scrambled (scr.) siRNA or *Ccne1* siRNA. Cells were harvested after 10 days and characterized by qPCR. **H** Gene expression analysis of *Ccne1* in *Cdk2*^f/f^ and *Cdk2*^ΔHSC^ mice after siRNA transfection showed approximately 90% knockdown efficiency of *Ccne1* siRNA. **I** Growth analysis of *Cdk2*^f/f^ (left panel) and *Cdk2*^ΔHSC^ HSCs transfected with either scrambled (scr.) or *Ccne1* siRNA. Analysis was performed as described for panel (**B**). **J**–**M** Gene expression analysis of **J** *Acta2* (left), *Col1a1* (right), **K** *Il6*, **L** *Pdgfrb*, and **M** *Ccl2*. All mRNA expression values were normalized to the expression of *Gapdh* and calculated as fold induction in comparison to either primary *Ccne1*^f/f^ HSCs (**C**–**F**) or *Cdk2*^f/f^ HSCs treated with scr. siRNA (**H**, **J**–**M**). Data are expressed as mean ± SD. *p ≤ 0.05; **p ≤ 0.005; ***p ≤ 0.001.

We next aimed to directly evaluate the impact of *Ccne1* and *Cdk2* on the pro-fibrotic properties of HSCs. To this end, we isolated primary HSCs from *Ccne1*^ΔHSC^ and *Cdk2*^ΔHSC^ mice and studied their in vitro viability and pro-fibrotic gene regulation in comparison to cre-negative control cells for a period of 10 days (Fig. 6A). Of note, the cell number of both primary *Ccne1*^f/f^ and *Cdk2*^f/f^ control HSCs decreased over time. However, cell number (and thus survival) of both *Ccne1*- and *Cdk2*-depleted HSCs was substantially lower and reached only approximately 50 % of density from matching WT control cells after 10 days of cultivation (Fig. 6B). This suggests that loss of *Ccne1* and *Cdk2* both results in significantly impaired survival of HSCs. As expected, the cre-mRNA expression could be detected in *Ccne1*^ΔHSC^ and *Cdk2*^ΔHSC^ HSCs, but not in floxed control cells (Supplementary Fig. 5A). Accordingly, *Ccne1* was undetectable in *Ccne1*^ΔHSC^ cells, but clearly expressed in *Ccne1*^f/f^, *Cdk2*^f/f^, and *Cdk2*^ΔHSC^ HSCs. In addition, we could also verify the lack of *Cdk2* expression in *Cdk2*^ΔHSC^ HSCs (Supplementary Fig. 5B). Immunofluorescence microscopy indicated strongly reduced activation (i.e., αSMA and Ki-67 expression) of *Ccne1*^ΔHSC^ and *Cdk2*^ΔHSC^ HSCs compared to WT (i.e., *Ccne1*^f/f^, *Cdk2*^f/f^) cells (Supplementary Fig. 5C), which was further confirmed by qPCR analysis of *Acta2* and *Col1a1* gene expression (Fig. 6C). Importantly, we found induction of *Il6* gene expression in WT HSCs after 10 days of cultivation, which was reduced almost to background levels by deletion of *Ccne1*, but not through inactivation of *Cdk2* (Fig. 6D). This suggests that *Ccne1* is involved in controlling IL6 secretion of HSCs in the pro-carcinogenic liver environment. Moreover, *Ccne1* and *Cdk2* were both required for the induction of *Pdgfrb* gene expression in HSCs during spontaneous in vitro activation (Fig. 6E) and for upregulation of the *Ccl2* gene (encoding the chemoattractant chemokine CCL2) in HSCs (Fig. 6F). In summary, these experiments clearly demonstrate that *Ccne1* is essential for growth, survival, activation and secretory functions of HSCs, which are mostly *Cdk2*-dependent (activation, PDGFR-β, CCL2), while expression of *Il6* is controlled by a novel *Cdk2*-independent function of *Ccne1*.

Next, we were interested to test whether concomitant inactivation of *Ccne1* and *Cdk2* in HSCs might result in synergistic effects. For this purpose, we encapsulated small interfering RNA (siRNA) specific for *Ccne1* in lipid nanoparticles (LNP) and transfected primary *Cdk2*^*f*/f^ or *Cdk2*^ΔHSC^ HSCs as illustrated in Fig. 6G. Using this approach, *Ccne1* gene expression was reduced to approximately 90% in both HSC groups (Fig. 6H). As a control, cells were transfected with a non-coding scrambled siRNA. In agreement with the data from genetic *Ccne1* inactivation (compare Fig. 6B), knockdown of *Ccne1* by siRNA in WT (i.e., *Cdk2*^*f/f*^) HSCs also resulted in a significant reduction of cell number over time. However, concomitant inhibition of *Ccne1* and *Cdk2* in *Ccne1* siRNA treated *Cdk2*^ΔHSC^ HSCs had no significant effect on this reduced viability (Fig. 6I). Concomitant inactivation of *Cdk2* and *Ccne1* reduced gene expression levels of *Acta2* and *Col1a1* in HSCs to the same extend as deletion of *Cdk2* alone (Fig. 6J). Importantly, these experiments confirmed that inhibition of *Ccne1* triggers down-regulation of *Il6* gene expression in a Cdk2-independent manner, which could be achieved by *Ccne1* siRNA delivery in both *Cdk2*^*f*/f^ or *Cdk2*^ΔHSC^ HSCs (Fig. 6K). Finally, combined inactivation of *Cdk2* and *Ccne1* down-regulated *Pdgfrb* and *Ccl2* gene expression to the same extent as deletion of *Cdk2* alone or *Ccne1* siRNA delivery alone (Fig. 6L, M). Taken together, these experiments demonstrate that *Ccne1* and *Cdk2* jointly control the expression of several pro-fibrotic genes in HSCs, whereas *Il6* expression is regulated by *Ccne1* in a Cdk2-independent manner.

**Fig. 7:**
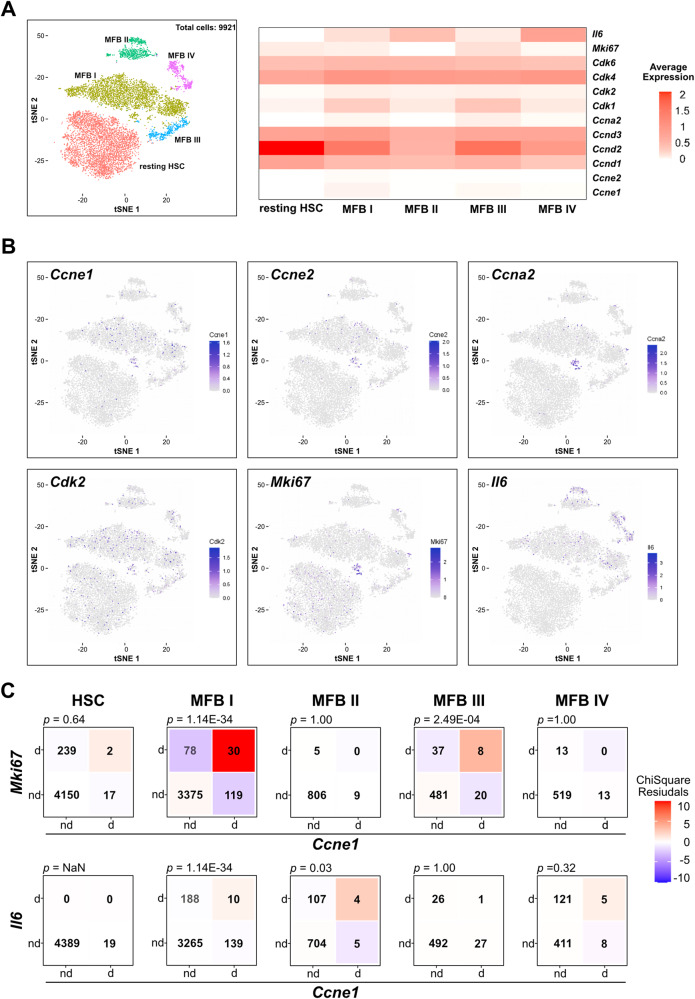
Identification of activated HSCs with an expression of *Ccne1* and *Cdk2* after chronic CCl_4_ treatment by single-cell RNA sequencing (scRNAseq). Expression analysis of cell cycle-related genes from single-cell sequencing data (scRNAseq) of resting and activated HSCs (i.e., myofibroblasts; MFB). **A** Left: Subset clustering of resting HSCs (HSC) and four different MFB sub-clusters referred to as MFB I–MFB IV from scRNAseq datasets serving as an overview. Data were adapted from [21]. Right: Heatmap showing average expression of G_1_/S-phase genes, *Mki67* and *Il6* for each cluster. **B** t-SNE plots show the relative gene expression strength of selected cell cycle genes and *Il6*. **C** Analysis of *Ccne1* co-expression with *Mki67* and *Il6* expression on single cell level. Upper panel: Expression of *Ccne1* is associated with *Mki67* expression in MFB I and MFB III clusters. Detection (>0 reads per cell) for *Ccne1* and *Mki67* were assessed for each cluster. Numbers in each tile denote numbers of cells; d: detected; nd: not detected; color shows Chi-Square-residuals of observed *versus* expected counts. *P*-values represent a Chi-square test of independence between the detection of the two genes. Lower panel: Expression of *Ccne1* is slightly associated with Il6 expression solely in cluster MFB I. Detection (>0 reads per cell) for *Ccne1* and *Il6* was assessed for each cluster. Color code and calculation were performed as above.

We finally aimed to evaluate the expression of *Ccne1* and related cell cycle genes in resting and activated HSCs during fibrogenesis on a single cell level. To this end, we took advantage of single-cell RNA sequencing (scRNAseq) data that we had generated before [21]. In that study, activated HSCs (i.e., myofibroblasts; MFB) were isolated from WT mice after chronic treatment with CCl_4_ for 3 weeks and subjected to scRNAseq. As a control, resting HSCs were isolated from untreated mice and processed accordingly. Due to different expression profiles, the cells were separated into five sub-clusters referred to as MFB I to MFB IV as well as resting HSC (Fig. 7A). We analyzed these data with respect to G_1_/S phase cyclins and Cdks. As our present experiments demonstrated that *Il6* is regulated by *Ccne1* in HSCs, we also investigated *Il6* expression in the scRNAseq data set.

Cell-cycle-dependent expression of *Ccne1* is typically restricted to the G_1_/S-phase transition [22, 23]. However, in resting HSCs or MFBs, *Ccne1* expression was only detected in 2.2% of all cells. *Ccne1* was mostly expressed in cells belonging to the MFB I cluster (Fig. 7B, C). Similarly, *Cdk2* (detected in 4.4% of all cells), *Ccne2* (detected in 1.3% of all cells), and *Ccna2* (detected in 1.6% of all cells) were also, at best, slightly expressed in only a small proportion of HSCs and MFBs (Fig. 7B, C). In good agreement, the number of *Mki67*-expressing cells (3.1%) was in a similar range in MFBs suggesting that the percentage of proliferating MFBs during fibrogenesis at a distinct time point is rather moderate. The expression profile of each individual cell with respect to cell cycle genes is depicted in Supplementary Fig. 6A, B. Surprisingly, we detected a strong gene expression of D-type cyclins and their interacting kinases *Cdk4*/*Cdk6* throughout all HSC/MFB clusters, while *Cdk1* was rather low expressed (Fig. 7A and Supplementary Fig. 6A–C).

We next assessed the expression of *Ccne1* and *Mki67* for each cluster and found a significant association of both genes in MFB I and MFB III (Fig. 7C, upper panel), suggesting that *Ccne1* mediates also in MFBs cell cycle-dependent functions. *Il6* expression was detected in only 8.4 % of the cells throughout cluster MFB I–MFB IV (Fig. 7B), lacking significant association with *Ccne1* expression except for MFB II where, however, *Ccne1* expression was mostly absent (Fig. 7C, lower panel).

In summary, the scRNAseq data suggest that the strong effects of the HSC-specific *Ccne1* deletion regarding liver fibrogenesis and HCC development are caused by a small number of actively cycling HSCs and MFBs. The data further implicate that the effects of *Ccne1* on *Il6* expression in HSCs might be indirect, as a significant co-expression in the same cells could not be verified.

## Discussion

HCC is primarily characterized by inappropriate, excessive proliferation of transformed hepatocytes/hepatoma cells. However, besides intrinsic aberrations within hepatocytes, such as de-regulation of cell cycle activators (e.g., Cyclins) or mutations of tumor suppressors (e.g., p53), also extrinsic signals from the tumor environment can contribute to HCC progression. The HCC tumor environment typically consists of cellular components, including tumor-associated macrophages, endothelial cells, immune cells, and activated HSCs, as well as non-cellular components, such as growth factors and extracellular matrix proteins [24]. In previous work, we have demonstrated that the cell cycle mediator *Ccne1* is essential for both initiation of liver fibrosis and HCC [6, 7, 10]. However, so far, we either constitutively deleted *Ccne1* in all cells or systemically inhibited *Ccne1* by siRNA, thereby targeting at least hepatocytes, HSCs, CD45^+^ cells, and endothelial cells to prevent fibrogenesis [7]. With regard to HCC, we have so far demonstrated that ubiquitous genetic inactivation of *Ccne1* or hepatocyte-specific deletion of its interacting kinase subunit *Cdk2* inhibited HCC initiation [10], while interventional depletion of *Ccne1*, but not of *Cdk2*, in hematopoietic cells, hepatocytes and lymphocytes ameliorated the progression of HCC [20]. Thus, our previous data does not exclude any of the main hepatic cell types from being a *Ccne1*-dependent driver of liver fibrosis or HCC.

Here, we tested the hypothesis that HSCs are important effector cells for *Ccne1*-driven liver fibrosis and hepatocarcinogenesis. To this end, we generated HSC-specific *Ccne1* knockout mice and challenged these animals with a pure liver fibrosis model (CCl_4_) as well as with a model reflecting both liver fibrogenesis and hepatocarcinogenesis (DEN/CCl_4_). Our study revealed that deletion of *Ccne1* only in HSCs substantially reduced liver fibrosis in both experimental settings, which was associated with reduced HSC activation. Importantly, the lack of *Ccne1* in HSCs also ameliorated HCC initiation and progression. Mechanistically, we demonstrated that the pro-fibrotic effect of *Ccne1* in HSCs is dependent on its associated kinase *Cdk2*. Finally, deletion of *Ccne1* in HSCs was associated with reduced expression of genes encoding important signaling molecules such as IL-6, PDGFR-β, and CCL2, which could be an additional key to explain the pro-fibrotic properties of *Ccne1*.

**Fig. 8:**
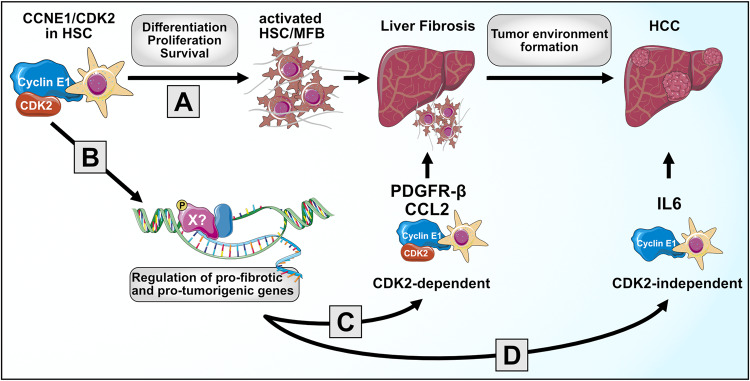
Proposed role of *Ccne1* and *Cdk2* for liver fibrogenesis and fibrosis-induced HCC. **A** Our data demonstrate that *Ccne1* and *Cdk2* are both required for the differentiation, proliferation, and survival of HSCs. Accordingly, the inactivation of *Ccne1* or *Cdk2* in HSCs reduces the pro-fibrotic properties of HSCs, thereby attenuating fibrosis formation upon chronic injury such as the CCl_4_ challenge. In turn, attenuated fibrosis may impact the tissue environment resulting in less liver tumor formation. **B** *Ccne1* in HSCs triggers liver fibrogenesis or HCC formation directly through transcriptional control of pro-fibrotic or hepatocarcinogenic signaling molecules. **C** The latter mechanism can be *Cdk2*-dependent by driving the expression of genes such as *Pdgfrb* (encoding PDGF-Rβ) and *Ccl2* (encoding CCL2). **D** We demonstrated that *Ccne1* is involved in transcriptional control of *Il6* in a *Cdk2*-independent manner, which is likely to contribute to HCC formation.

Thus, our study demonstrates that *Ccne1* is an important modulator of HSCs in the course of liver fibrosis and cancer development. This is especially remarkable as HSCs represent only 5–8% of total liver cells [2], and only a few of them (approximately 2%) showed *Ccne1* expression at a distinct point in time in our scRNAseq analyses when chronically challenged with CCl_4_. It must be pointed out that *Ccne1* is only expressed at the G_1_/S-phase transition and is degraded immediately afterward [25]. The duration of the complete S-phase in HSCs is not precisely known; however, in rat hepatocytes, it has been estimated that S-phase lasts approximately 7 h [26]. Thus, our scRNAseq measurements regarding *Ccne1* may reflect only a snapshot of (non-synchronized) HSCs that underwent S-phase initiation exactly at the moment of isolation. It is, therefore, very likely that in a longer observation period, many more HSCs will express *Ccne1* once. The number of *Ccne1*-expressing cells detected by scRNAseq was very similar to the number of *Cdk2*-expressing HSCs and correlated with *Mki67*-expressing HSCs, implicating that one function of *Ccne1* is indeed to drive the HSC cell cycle, in a *Cdk2*-dependent manner. This is further supported by our observations in primary HSCs confirming that proliferation and survival of HSCs require both *Ccne1* and *Cdk2*. Our current hypothesis on the role of *Ccne1* in HSCs is schematically shown in Fig. 8. Accordingly, poor survival and reduced proliferation capacity of HSCs due to *Ccne1* or *Cdk2*-deficiency is one possible mechanism to explain the substantial reduction of liver fibrosis in our study (Fig. 8A). Weakened fibrosis per se in combination with a reduced absolute number of activated HSCs could subsequently result in a modulation of the liver tissue environment that will act less tumorigenic upon a secondary oncogenic stimulation such as DEN treatment. Our conclusion is in perfect agreement with the very recent study of Filliol et al. [9], which shows in a variety of extensive experiments that activated HSCs overall have a tumor-promoting role in mouse models of HCC, including the DEN/CCl_4_ model. In this context, the authors of that study convincingly demonstrated that the depletion of HSCs in mice resulted in suppression of the DEN/CCl_4_-mediated HCC development [9]. Thus, it is logical that the reduction of HSC survival and activation (as observed in *Ccne1*^ΔHSC^ mice) should also result in reduced HCC load after DEN/CCl_4_-treatment. In addition, our data suggest that gene expression/transcription of several pro-inflammatory/pro-carcinogenic signaling molecules is directly controlled by *Ccne1/*CCNE1 (Fig. 8B), and this effect is either *Cdk2*-dependent (e.g., in case of *Pdgfrb* and *Ccl2*, Fig. 8C) or *Cdk2*-independent in case of *Il6* gene expression (Fig. 8D). Of note, this finding has not only been proven in a genetic approach (using gene deletion) but also in knockdown experiments using LNP-encapsulated *Ccne1* siRNA, pointing to therapeutic applicability of our results.

The mechanism by which CCNE1 alone or in combination with CDK2 controls the transcription of pro-fibrotic genes is unclear to date. However, there is overwhelming evidence that CCNE1/CDK2 can indeed control the transcription of target genes *via* several different mechanisms, as outlined elsewhere in detail [27]. Already as part of its canonical function, CCNE1/CDK2 enables the transcription of E2F-target genes by phosphorylation of the retinoblastoma protein RB. More recent studies suggest that CCNE1/CDK2 is also involved in the modulation of several other transcriptional regulators, such as Id2 [28], Myc [29], or NF-Y [30] through phosphorylation. We, therefore, conclude that CCNE1 alone or in complex with CDK2 can, in general, act as a transcriptional co-factor, and we postulate that this may also be true for the control of some pro-fibrotic genes, although the exact targets of this mechanism have to be evaluated in extensive future studies.

PDGF is the most potent pro-fibrotic factor associated with HSC proliferation and activation. Overexpression of its receptor PDGFR-β in the liver can be linked with enhanced fibrogenesis, steatosis, and HCC in mice [31]. In our study, HSC-specific deletion of *Ccne1* was associated with the downregulation of *Pdgfrb* gene expression in the DEN/CCl_4_ model in vivo but also in primary HSCs in vitro. This suggests that the downregulation of *Pdgfrb* in the DEN/CCl_4_-treated liver of *Ccne1*^ΔHSC^ mice is best explained by a direct function of *Ccne1* on *Pdgfrb* transcription and not by a simple reduction of the absolute HSC number in the injured liver. Of note, *Lrat*-Cre-mediated deletion of *Pdgfrb* inhibited HSC activation, liver fibrosis, and tumorigenesis in *Mdr2*^−*/−*^ mice in the study of Filliol et al. [9], which further suggests that down-regulation of PDGFR-β transcription by *Ccne1* inhibition is mechanistically related to reduced fibrogenesis and hepatocarcinogenesis in our own experimental setting.

Furthermore, our data demonstrated that also the chemokine CCL2 is transcriptionally regulated by *Ccne1* in a *Cdk2*-dependent fashion both in DEN/CCl_4_-treated liver and in primary HSCs. CCL2 regulates the migration and infiltration of monocytes/macrophages through combination with its specific receptor CCR2 and has a vital role during liver fibrogenesis [32]. Downregulation of *Ccl2* in *Ccne1*^ΔHSC^ mice could explain the reduced numbers of CD11b^+^ cells observed in these mice. These results indicate that *Ccne1* in HSCs can modulate the tumor microenvironment through CCL2 and also suggests that the interaction of HSCs and TAMs may have an important impact on tumor development and progression, as proposed recently [32]. Our study also revealed that *Ccne1* regulates *Il6* gene expression in HSCs independent of *Cdk2*. It has been previously shown that IL6 is essential for proper DEN-induced hepatocarcinogenesis in mice, and Kupffer cells within the liver have been identified as a major source of HCC-inducing IL6 [33]. However, a recent paper detected considerable IL6 expression also in HSCs. In turn, HSC-derived IL6 was able to promote HCC progression in mice through the induction of myeloid-derived suppressor cells (MDSCs), thereby modulating the tumor environment. Of note, the ablation of *Il6* gene expression in HSCs substantially attenuated HCC growth in that study [34]. In our work, we detected reduced *Il6* expression in the fibrotic liver of DEN/CCl_4_-treated *Ccne1*^ΔHSC^ mice, but also in *Ccne1*-deficient HSCs in vitro, while ablation of *Cdk2* in HSCs had no significant effect on *Il6* regulation. We, therefore, conclude that the down-regulation of *Il6* through *Ccne1*-depletion in HSCs is significantly involved in the reduced tumor burden of DEN/CCl_4_-treated *Ccne1*^ΔHSC^ mice.

Our scRNAseq analyses detected *Il6* gene expression only in a few HSCs following CCl_4_ treatment. Of note, the frequency of *Il6*-expressing HSCs was very similar to the number of HSCs with detectable *Ccne1* expression. However, significant co-expression of *Ccne1* and *Il6* could at a distinct time point was almost undetectable on a single cell level. We, therefore, assume that *Ccne1* induces *Il6* in HSCs either with a time delay or indirectly involving cell-cell communications.

Our final conclusion is summarized in Fig. 8: *Ccne1* in HSCs is an important modulator of fibrosis-driven HCC through at least 3 mechanisms by (i) enabling proliferation and survival of HSCs, (ii) controlling *Cdk2*-dependent expression of oncogenic signaling molecules, and (iii) driving *Cdk2*-independent *Il6* expression. Of note, ubiquitous deletion of *Ccne1* was similarly effective in preventing liver fibrosis [6] but much more potent in inhibiting HCC initiation [10] when compared to HSC-specific *Ccne1* inhibition. Thus, HSCs are the main effector cells for the pro-fibrotic function of *Ccne1*, but presumably only one out of several effector cells for the oncogenic function of *Ccne1*. Nevertheless, our data strongly indicate that therapeutic *Ccne1* inhibition will be beneficial for the treatment of both liver fibrosis and fibrosis-induced HCC.

## Material and methods

### Maintenance of mice and general animal experimentation

Animals were housed in a temperature-controlled room with 12-h light/dark cycles and free access to food and water. For this study, we crossed conditional *Ccne1* (*Ccne1*^f/f^) or *Cdk2* (*Cdk2*^f/f^) knockout mice [35, 36] with transgenic mice expressing Cre-recombinase under the control of the *L-rat* promoter [37], resulting in mice lacking *Ccne1* or *Cdk2* specifically in HSCs (*Ccne1*^∆HSC^, *Cdk2*^∆HSC^). For experiments, we used male mice in a C57BL/6 background; floxed (f/f), cre-negative littermates served as controls.

For induction of liver fibrosis, 8-week-old mice were injected with CCl_4_ (0.5 ml/kg *i.p*., diluted in corn oil) three times a week for 6 consecutive weeks. For induction of liver fibrosis and HCC, we applied the DEN/CCl_4_ model in which fibrosis is induced by a single injection of *N*-nitrosodiethylamine (DEN) followed by repeated administration of carbon tetrachloride (CCl_4_) following a protocol previously described [12] with minor modifications. Briefly, 14-day-old pups were injected with DEN (25 mg/kg*, i.p*.), followed by weekly injections of CCl_4_ (0.5 ml/kg, diluted in corn oil) for 18 weeks, starting at the age of 6 weeks. All animals were euthanized 48 h after the last CCl_4_ injection by cervical dislocation.

### Tumor quantification

Livers were removed and analyzed for the presence of visible tumors. Tumor-bearing livers were documented using a Leica MZ16 microscope together with Diskus software (Hilgers, Königswinter, Germany). Separation into individual lobes was followed by measuring the diameter of visible surface nodules in millimeters. Intrahepatic tumors were identified and counted on Hematoxylin & Eosin (H&E) stained liver sections. Each liver section was completely scanned using an Axio Observer.Z1 microscope (Carl Zeiss, Jena, Germany), followed by quantification of all tumor nodules and of the tumor-free area using Image J software (http://rsbweb.nih.gov/).

### Isolation and cultivation of primary HSCs

Primary mouse HSCs were isolated from 28 to 35-week-old mice by collagenase perfusion as described recently [6] with the modification that animals were sacrificed before cell isolation. After isolation, living cells were plated on 12-well cell+ plates (Greiner, Kremsmünster, Austria) at a density of 30,000 cells per 12-well in DMEM medium (PAA Laboratories GmbH, Pasching, Austria) supplemented with 10% FCS and 100 U/ml penicillin/streptomycin. After 4 h, cells were attached, and dead cells were removed by washing with PBS and medium exchange. Each day, the same area of the plate was imaged using an Axio Imager.Z1 microscope (Carl Zeiss) and analyzed for cell density.

### Generation of lipid nanoparticles and transfection of primary HSCs with siRNA

The used siRNAs were ordered from IDT (Leuven, Belgium) in a lyophilized form and resolved in 0.1 M acetate buffer. Sequences of used siRNAs are listed in Supplementary Table 2.

For generation of LNPs, the lipids Cholesterol, 1,2-distearoyl-sn-glycero-3-phosphocholine (DSPC), and 1,2-Dimyristoyl-rac-glycero-3-methoxypolyethylene glycol-2000 (DMG-PEG 2000) were acquired from Sigma Aldrich (St. Louis, MO). Heptatriconta-6,9,28,31-tetraen-19-yl-(dimethylamino)butanoate (DLin MC3-DMA, MC3) was obtained from Hycultec (Beutelsbach, Germany). Lipids were resolved in absolute ethanol at the molar ratios of 50:10:38.5:1.5 (MC3:DSPC:Cholesterol:DMG-PEG2000). LNP were generated using a NanoAssemblr Spark (Precision Nanosystems, Vancouver, Canada) according to the manufacturer’s instructions. For larger-scale production, we performed LNP generation using two Ph.D. Ultra syringe pumps (Harvard Apparatus, Holliston, MA) connected to a Herringbone microchip (Microfluidics ChipShop, Jena, Germany) and mixing was done at a flow rate ratio of 3:1 (siRNA:lipids). LNP were characterized for size using dynamic light scattering (DLS) and surface charge using Zetapotential (Malvern Zetasizer, Malvern, UK). A Ribogreen assay was done to quantify the RNA content of LNP, as published before [38].

For transfection, primary HSCs were seeded on Petri dishes immediately after isolation and cultivated for 4 days. Immediately before transfection at day 4, HSCs were washed once with PBS and supplied with fresh medium containing siRNA at a concentration of 1 µg/ml encapsulated in LNPs as described above. Transfected HSCs were then cultivated for another 6 days without further treatment.

### Single-cell RNA sequencing (scRNAseq) and bioinformatics data analysis

For analysis of HSC gene expression on a single cell level, we used recently published data [21]. In the underlying experiments, wild-type (WT) mice were treated 3 times a week with CCl_4_ for 3 weeks and sacrificed 36 h after the last treatment. Activated HSCs (i.e., myofibroblasts; MFB) were isolated from these animals and subjected to scRNAseq. As a control, resting HSCs were isolated from untreated mice and processed accordingly. Cells were analyzed by using the Chromium Single Cell 50 kit (10× Genomics, Pleasanton, CA, USA), according to the manufacturer’s protocol. After library generation, sequencing was performed by Illumina sequencing on a NextSeq 550. The primary analysis was done by using an in-house pipeline based on “cellranger” (10× Genomics). Additional analysis was then performed by using the “Seurat” (v2.3.2) package for R (v3.5) (https://www.r-project.org/).

### Statistical analysis

For statistical analysis of the results, Graph Pad Prism 8 (Graphpad Software, Inc., USA) was used. All results were first tested for normal distribution using the Shapiro-Wilk test. Comparison of two groups was performed by an unpaired, two-tailed t-test in case of a normal distribution or by Mann Whitney test in case of non-normal distributed results. Comparison of three or more groups differing in one variable (e.g., genotype) was performed using ordinary one-way ANOVA with Tukey’s multiple comparisons test. In the case of groups differing in two variables (e.g., genotype and treatment), two-way ANOVA with Tukey’s multiple comparisons test was performed. All data were presented as mean ± standard deviation (SD) using combined dot plots with bars to indicate values of individual mice or biological replicates. Significances were defined as *p ≤ 0.05; **p ≤ 0.005; ***p ≤ 0.001.

## Supplementary information

Supplementary Material

CDD-checklist

## Data Availability

Raw expression data of single-cell sequencing datasets used within this study is deposited in the Gene Expression Omnibus (GEO, see https://www.ncbi.nlm.nih.gov/geo/) under accession No. GSE132662. The main raw data on which the study is based will be made available by the corresponding author upon reasonable request.
