# Supplementary material for: Expression of Cyclin E1 in hepatic stellate cells is critical for the induction and progression of liver fibrosis and hepatocellular carcinoma in mice
Source: Cell Death Dis. 2023 Aug 24;14(8):549. doi: 10.1038/s41419-023-06077-4 (PMC10449804; doi:10.1038/s41419-023-06077-4)
Supplement: Supplementary file 1 — Supplementary Material [file 41419_2023_6077_MOESM1_ESM.pdf]

Expression of Cyclin E1 in Hepatic Stellate Cells is critical for induction and progression of liver fibrosis and hepatocellular carcinoma in mice

Julia Otto, Anna Verwaayen, Christian Penners, Jana Hundertmark, Cheng Lin, Carina Kallen, Daniela Paffen, Tobias Otto, Hilmar Berger, Frank Tacke, Ralf Weiskirchen, Yulia A. Nevzorova, Matthias Bartneck, Christian Trautwein, Roland Sonntag, and Christian Liedtke

Supplementary Material

Supplementary Figures and Legends

- Supplementary Fig. 1 *Ccne1*$^{\Delta HSC}$ mice do not display an obvious liver phenotype under basal conditions
- Supplementary Fig. 2 Strength of liver fibrosis significantly correlates with tumor load after DEN/CCl_4 treatment in *Ccne1*$^{\Delta HSC}$ mice
- Supplementary Fig. 3 Characterization of proliferating cell populations in DEN/CCl_4 treated murine livers with fibrosis and HCC.
- Supplementary Fig. 4 Loss of Cyclin E1 in HSCs does not affect populations of hepatic NK, NKT, B- and T-cells during hepatocarcinogenesis.
- Supplementary Fig. 5 Activation of primary HSCs is reduced by deletion of *Ccne1* or *Cdk2*, as indicated by decreased αSMA expression and proliferation.
- Supplementary Fig. 6 Analysis of resting and activated HSCs for expression of D-type cyclins and *Cdk4/6* after chronic CCl_4 treatment by scRNAseq

Supplementary Material and Methods

- Determination of serum transaminase activities
- Liver histology, Sirius red staining and quantification
- RNA isolation and quantitative Real-Time PCR (qPCR)
- Fluorescence activated Cell Sorting (FACS) of liver cells

Supplementary Tables

- Supplementary Table 1: Primer sequences used for qPCR
- Supplementary Table 2: siRNA sequences used for transfection of HSCs
- Supplementary Table 3: Fluorochrome-labeled antibodies used for FACS analysis
- Supplementary Table 4: Primary and secondary Antibodies used for Immunohisto-chemistry stainings

Supplementary References

SUPPLEMENTARY FIGURES

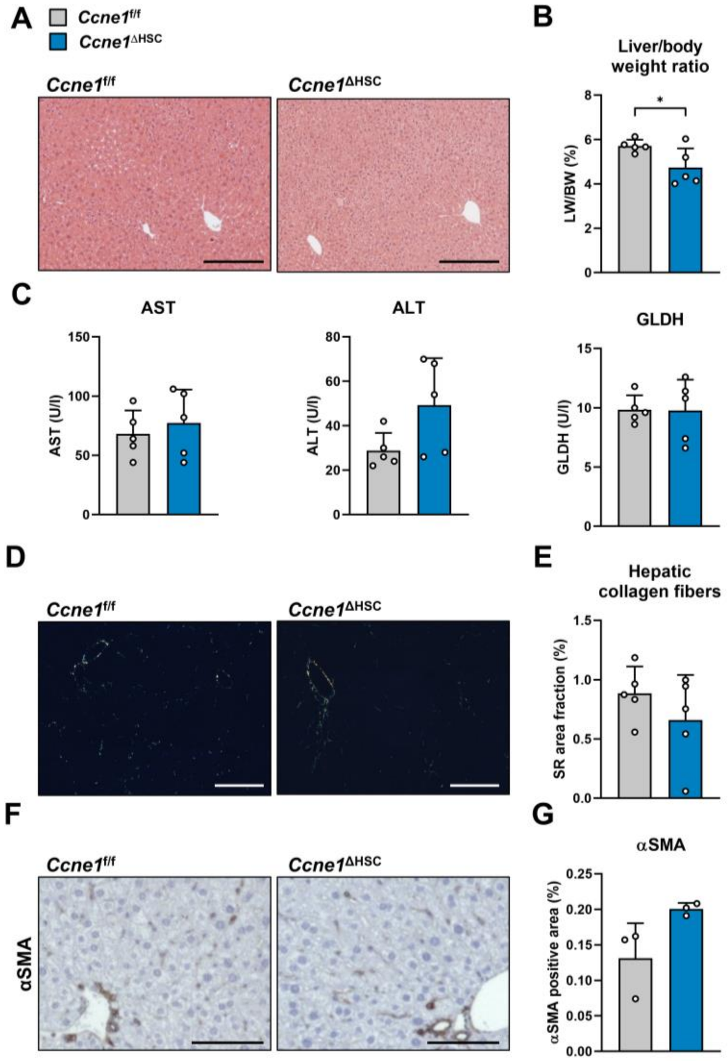

Supplementary Fig. 1 *Ccne1*$^{\Delta HSC}$ mice do not display an obvious liver phenotype under basal conditions. Untreated *Ccne1*$^{\Delta HSC}$ mice (n=5, blue bars) and *Ccne1*$^{f/f}$ (n=5, grey bars) littermates at the age of 24 weeks were investigated for markers of liver homeostasis, injury and pro-fibrogenic properties. **A.** Representative Hematoxylin and Eosin (H&E) - stained liver paraffin sections from *Ccne1*$^{f/f}$ and *Ccne1*$^{\Delta HSC}$ mice at the age of 24 weeks. Scale bar: 100 µm. **B.** Determination of relative liver mass index calculated as the liver weight (LW): body weight (BW) ratio given in percent. **C.** Determination of AST, ALT and GLDH activities. Values are given as Units/liter (U/l). **D.** Representative Sirius red stainings of liver sections. Images were taken with a polarizing filter; collagen fibers appear in orange. Scale bar: 100 µm. **E.** Morphometric quantification of basal liver collagen. Sirius Red stained images shown in D. were analyzed for Sirius Red-positive image areas (percentage of total tissue areas) using ImageJ software. **F.** Immunohistochemistry staining of αSMA. Scale bar: 100 µm. **G.** Morphometric quantification of αSMA positive areas from the IHC staining shown in **G**. using image J (IHC toolbox). Data are expressed as mean ± SD. *$p \leq 0.05$.

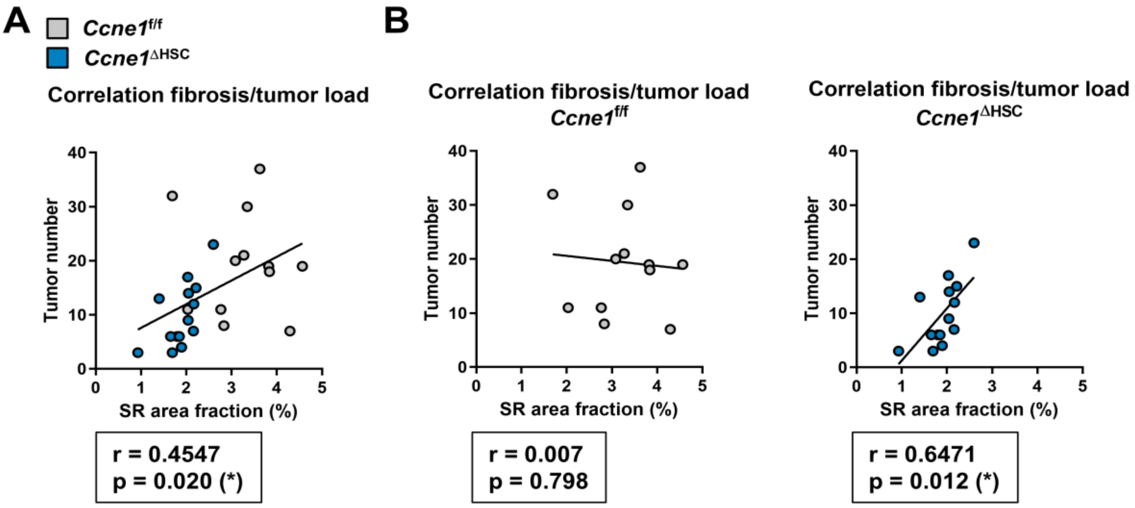

Supplementary Fig. 2 Strength of liver fibrosis significantly correlates with tumor load after DEN/CCl_4 treatment in *Ccne1*$^{\Delta HSC}$ mice.

Ccne1$^{\Delta HSC}$ mice (n=15, blue dots) and *Ccne1*$^{f/f}$ littermates (n=12, grey dots) were subjected to the DEN/CCl_4 HCC model as illustrated in Figure 2A. Mice were sacrificed at the age of 24 weeks. Pearson correlation analysis was performed by plotting Sirius red (SR) area fractions of tissue sections (as a measure of liver fibrosis) against the number of macroscopic liver tumors (as a measure of tumor load) for each individual animal. r: Pearson correlation coefficient. **A.** Pearson correlation of all investigated mice. **B.** Pearson correlation investigated in the group of control mice (left) or *Ccne1*$^{\Delta HSC}$ mice (right). *: $p \leq 0.05$.

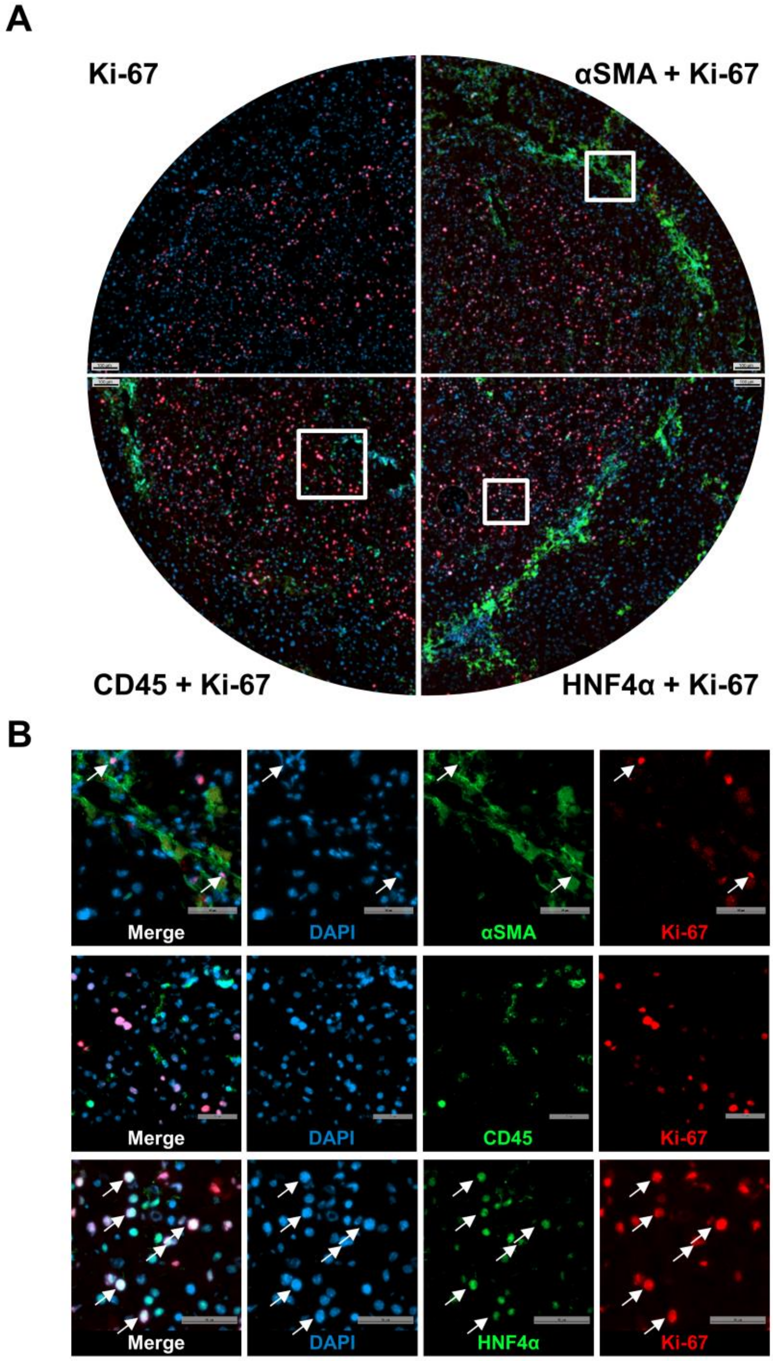

Supplementary Fig. 3 Characterization of proliferating cell populations in DEN/CCl_4 treated murine livers with fibrosis and HCC.

Ccne1$^{f/f}$ (*i.e.* WT) mice were subjected to the DEN/CCl_4 HCC model as illustrated in Figure 2A. Liver cryosections of animals with proven fibrosis and HCC were co-stained for Ki-67 and a cell type specific marker for either HSCs (*i.e.* αSMA), immune cells (*i.e.* CD45) or hepatocytes (*i.e.* HNF4α). **A.** Representative overview of a liver section including a tumor stained with Ki-67 alone (red) or in combination with one of the indicated cell type specific markers (green). Co-stainings were performed on serial sections of frozen tissue followed by image acquisition of the same tissue region. **B.** Enlargement of sections marked in A. Arrows indicate representative double positive cells. Scale bar: 50 µm.

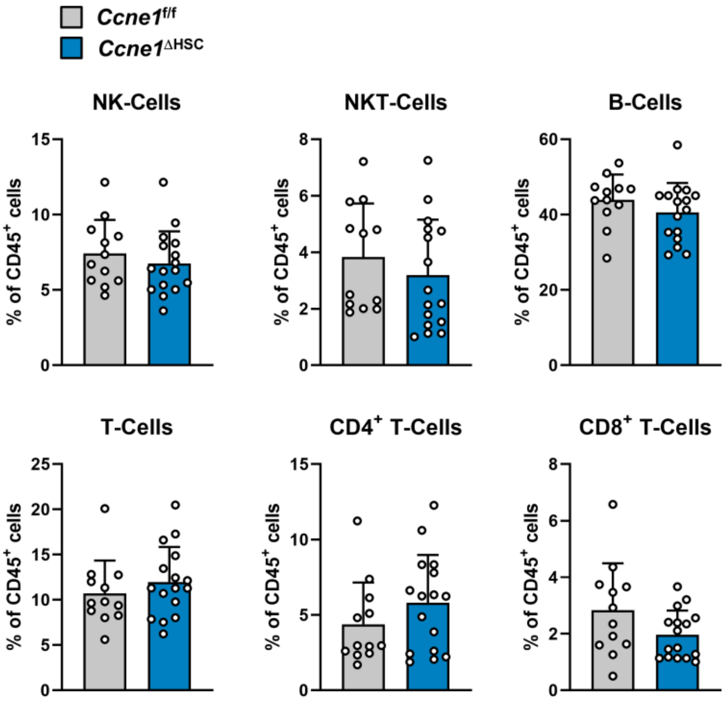

Supplementary Fig. 4 Loss of Cyclin E1 in HSCs does not affect populations of hepatic NK, NKT, B- and T-cells during hepatocarcinogenesis.

Ccne1$^{\Delta HSC}$ (n=15, blue bars) and *Ccne1*$^{f/f}$ mice (n=12, grey bars) were challenged with DEN and CCl_4 as described before to induce liver fibrosis and HCC. At the age of 24 weeks, mice were sacrificed. From explanted livers, single cell suspensions were isolated and subjected to FACS analysis. Hepatic immune cell populations were identified by cell-type specific surface markers (given in brackets) for NK ($CD3^-$, $NK1.1^+$), NKT ($CD3^+$, $NK1.1^-$), B ($CD3^-$, $CD19^+$), and T-cells ($CD3^+$). Subpopulations of T-cells were differentiated according to the expression of CD4 or CD8, respectively. Values are indicated as percentage of total CD45-positive cells. Data are expressed as mean ± SD.

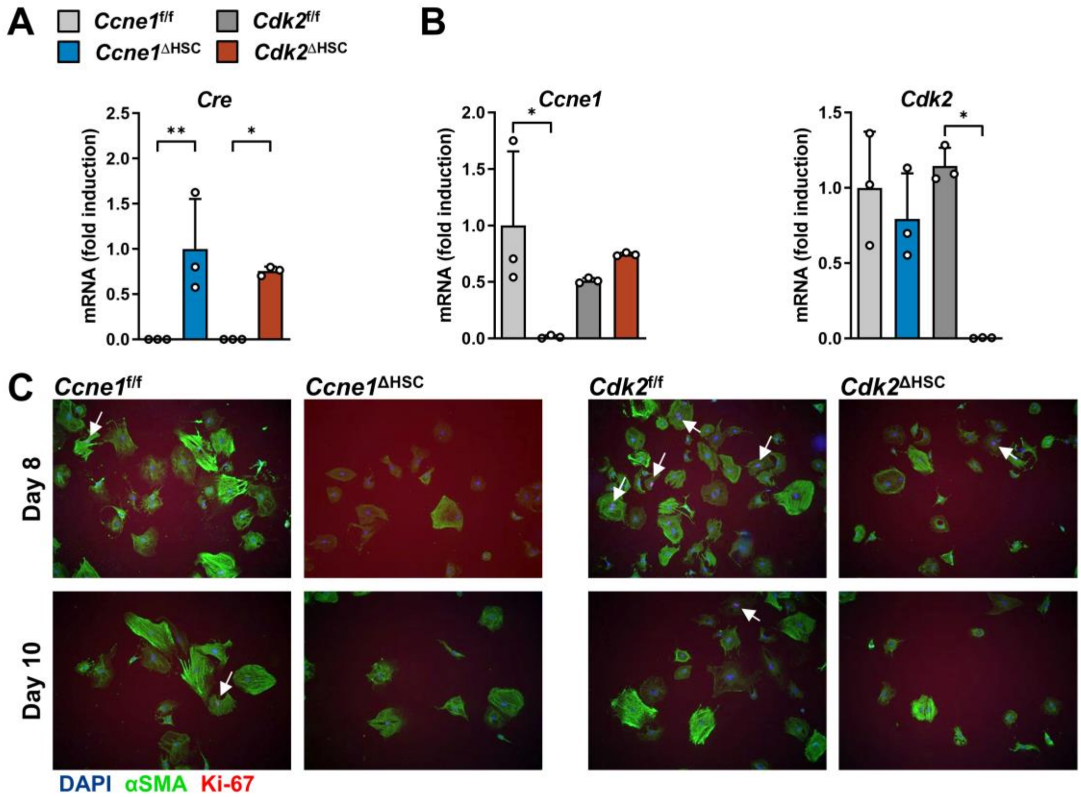

Supplementary Fig. 5 Activation of primary HSCs is reduced by deletion of *Ccne1* or *Cdk2*, as indicated by decreased αSMA expression and proliferation.

Primary HSCs were isolated from *Ccne1*$^{\Delta HSC}$ and *Cdk2*$^{\Delta HSC}$ mice as well as from respective cre-negative (*i.e.* *Ccne1*$^{f/f}$, *Cdk2*^{ff}) littermate controls. Cells were cultivated for up to ten days (D0 - D10) and analyzed at daily intervals. **A-B**. Gene expression analysis as determined by qPCR. **A**. Cre-recombinase (*Cre*), **B**. *Ccne1* (left), and *Cdk2* (right). Expression values were normalized to expression of *Gapdh* and calculated as fold induction in comparison to either primary *Ccne1*$^{\Delta HSC}$ HSCs (panel C) or *Ccne1*$^{f/f}$ HSCs (panel D) at day zero (D0). **C.** Primary HSCs were seeded on cover slips and harvested at the time points indicated. Cells were stained with fluorescence-labelled antibodies against αSMA (green) and Ki-67 (red, arrows) and analyzed by fluorescence microscopy. Data are expressed as mean ± SD. *$p \leq 0.05$; **$p \leq 0.005$.

A

Gene	total no. of cells with detection	% of total cells
Ccne1	218	2,2
Ccne2	129	1,3
Il6	462	4,7
Mki67	412	4,2
Cdk6	2602	26,2
Cdk4	5183	52,2
Cdk2	432	4,4
Cdk1	965	9,7
Ccna2	156	1,6
Ccnd3	4763	48,0
Ccnd2	7446	75,1
Ccnd1	2984	30,1

B

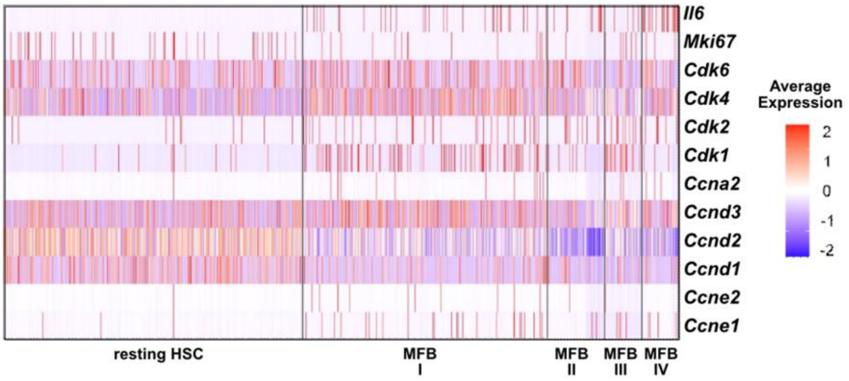

C

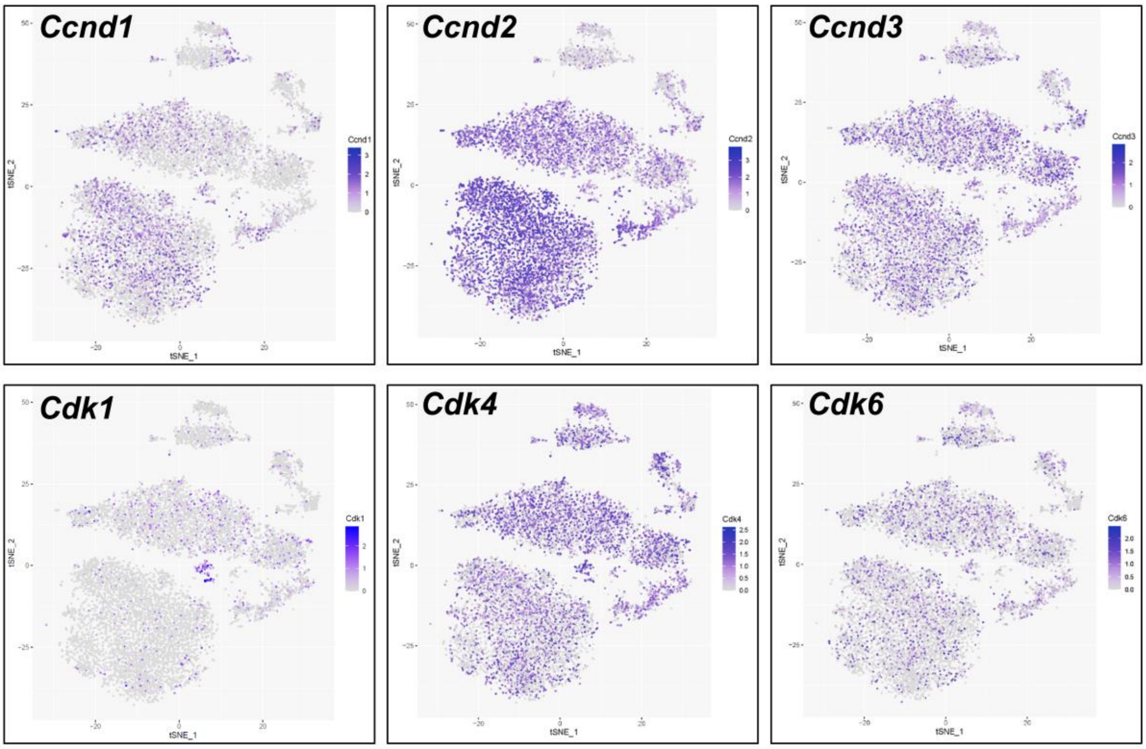

Supplementary Fig. 6 Analysis of resting and activated HSCs for expression of D-type cyclins and Cdk4/6 after chronic CCl_4 treatment by scRNAseq.

Expanded data related to Figure 7. Expression analysis of cell cycle related genes from single cell sequencing data (scRNAseq) of resting and activated HSCs (*i.e.* myofibroblasts; MFB). **A**. Absolute and relative cell counts of total HSCs/MFBs with detected expression of indicated G_1/S-phase genes and *Mki67*/*Il6*. **B**. Heatmap showing single cell expression of G_1/S-phase genes, *Mki67* and *Il6* for each cluster. **C.** t-SNE plots showing the relative gene expression strength of D-type cyclins and related *Cdk4* and *Cdk6*. Gene expression of *Cdk1* is also shown for comparison.

SUPPLEMENTARY MATERIAL AND METHODS

Determination of serum transaminase activities

Alanine aminotransferase (ALT), aspartate aminotransferase (AST) and glutamate dehydrogenase (GLDH) were determined in serum at the Laboratory Diagnostic Center (LDZ) of the RWTH University Hospital Aachen.

RNA isolation and quantitative Real-Time PCR (qPCR)

Total RNA from liver tissues or cell pellets was isolated using the peqGOLD RNAPureTMKit (Peqlab, Erlangen, Germany). Reverse-transcription was performed using an Omniscript RT Kit (Qiagen, Hilden, Germany). Relative quantitative gene expression was measured *via* real-time PCR using a 7300 Real Time PCR System with SDS software 1.3.1 (Applied Biosystems, Foster City, CA, USA) and a FastSYBR Green PCR Kit (Invitrogen, Carlsbad, CA, USA). Target gene expression was normalized to GAPDH expression as internal standard and calculated as fold induction in comparison to untreated controls. Primer sequences used in this study are listed in Supplementary Table 1.

Fluorescence activated Cell Sorting (FACS) of liver cells

FACS analysis of liver cells was basically performed as described before [1]. Briefly, mice were sacrificed and blood leukocytes were removed from liver tissue by perfusion with phosphate-buffered saline. After removal of the liver, 0.4 – 0.6 mg of liver tissue was digested with Collagenase II (Worthington, Lakewood, NJ, USA) at 37 °C. The digested liver suspension was filtered through a 70 µm cell strainer and remaining erythrocytes were lysed using BD Pharm Lyse buffer (BD Biosciences, San Jose, CA, USA). The resulting cell suspension was stained with fluorochrome conjugated antibodies specific for myeloid cells or lymphoid cells as listed in Supplementary Table 3. Analysis was performed using a FACS LSRFortessa Flow Cytometer (BD Biosciences, Heidelberg, Germany); data were analyzed using FlowJo software version 7.5 (FlowJo, Ashland, OR, USA). Cells were pre-gated on CD45 to identify

leukocytes. Dead cells were excluded by Hoechst 33258 incorporation and sub-gating was performed as described earlier with minor modifications [2, 3]. Total cells per liver were calculated using BD Calibrite APC beads for calibration.

Liver histology, Sirius red staining and quantification

Hepatic tissues were fixed in 4% paraformaldehyde (PFA) immediately after extraction, embedded in paraffin, sectioned and subjected to staining for Hematoxylin/Eosin (H&E) and Sirius red. The amount of Sirius red stained liver tissue area in all animals was quantified on recording of entire slides, with use of the NIH ImageJ software (http://rsbweb.nih.gov/) as described recently[4]. For the quantification of activated HSCs, paraffin liver sections were immunohistochemically stained with an antibody for alpha-smooth-muscle actin (αSMA).

Immunofluorescence staining of liver cryosections was performed according to our recent report [5]; used antibodies are listed in Supplementary Table 4. For staining of total nuclei, DAPI mounting medium (BD Bioscience, Franklin Lakes, NJ, USA) was used. For staining of primary HSCs, cells were seeded on cover slips and harvested at distinct time points. Microscopy, image acquisition and analysis were performed using an Axio Imager Z1 microscope and AxioVision software (Zeiss).

SUPPLEMENTARY TABLE 1: Primer sequences used for qPCR

Target gene	Sense primer (5′→3′)	Anti-sense primer (5′→3′)
Pdgfrb	TCAAGCTGCAGGTCAATGTC	CCATTGGCAGGGTGACTC
Ccne1	ACAGCAGGTCTTCGTGCAGATCG	CAGCGAGGACACCATAAGGAAATTC
Ccne2	AAAAAGTCTTGGGCAAGGTAAA	GCATTCTGACCTGGAACCAC
Col1a1	GGAACAGACGGCTGAGTAGG	TCTGACTGGAAGAGCGGAGAG
Gapdh	AACCTGCCAAGTATGACATCA	TGTTGAAGTCACAGGAGACAACCT
Acta2	TGACAGAGGCACCACTGAACC	TCCAGAGTCCAGCACAATACCAGT
Alb	TGTCCCCAAAGAGTTTAAAGCTG	TCTTAATCTGCTTCTCCTTCTCTGG
Prom1	CAAACCCATGGCCACCGCGA	CACCGTGGCTTTCCCTATGCCG
Afp	AGCAAAGCTGCGCTCTCTAC	AGGGGCTTTCCTCGTGTAAC
Mki67	CTTCTGTGCTGACCCTGATGG	CATCTGAGGCAGGGCTATCTG
Il6	GCTACCAAACTGGATATAATCAGGA	CCAGGTAGCTATGGTACTCCAGAA
Tnf	ACCACGCTCTTCTGTCTACTGA	TCCACTTGGTGGTTTGCTACG
Ccl2	GTGTTGGCTCAGCCGATGC	GACACCTGCTGCTGGTGATCC
Ccl5	TGCTGCTTTGCCTACCTCTC	TCCTTCGAGTGACAAACACGA
Cre	CTGACGGTGGGAGAATGTTAAT	TCGCTCGACCAGTTTAGTTACC
Cdk2	TCGGTGAAGGACACGGTGAG	TCATGGATGCCTCTGCTCTC

SUPPLEMENTARY TABLE 2: siRNA sequences used for transfection of HSCs

Target gene	Forward	Reverse
Ccne1	AUGAAAUUCUUACCAUGGAAUUGAT	CCUACUUUAAGAAUGGUACCUUAACUA
scrambled	CUUCCUCUCUUUCUCUCCCUUGUGA	UCACAAGGGAGAGAAAGAGAGGAAGG

SUPPLEMENTARY TABLE 3: Fluorochrome-labeled antibodies used for FACS analysis (MP – Myeloid panel, LP – lymphoid Panel)

Marker	Fluorochrome	Panel	Source
CD45	APC-Cy7	MP/LP	no. 47-0451-82; Thermo Fisher Scientific
GR1.1	PerCP-Cy5.5	MP	no. 552093; BD Biosciences
F4/80	PE-Cy7	MP	no. 25-4801-82; Thermo Fisher Scientific
CD11b	PE	MP	no. 12-0112-83; Thermo Fisher Scientific
MHCII	FITC	MP	no. 11-5321-82; Thermo Fisher Scientific
CD11c	APC	MP	no. 17-0114-82; Thermo Fisher Scientific
Ly6G	AL700	MP	no. 56-9668-82; Thermo Fisher Scientific
CD19	PerCP-Cy5.5	LP	no. 551001; BD Biosciences
NK1.1	PE-Cy7	LP	no. 25-5941-82; Thermo Fisher Scientific
CD4	PE	LP	no. 12-0041-83; Thermo Fisher Scientific
CD8	FITC	LP	no. 11-0081-85; Thermo Fisher Scientific
CD3	APC	LP	no. 12-0031-81; Thermo Fisher Scientific
Ki-67	AL700	LP	no. 56-5698-82; Invitrogen
Hoechst	V450	MP/LP	ab228551; BD Bioscience

SUPPLEMENTARY TABLE 4: Primary and secondary antibodies used for *in situ* tissue stainings

Marker	Source
Ki-67	14-5698-82; Invitrogen
αSMA	#2547; Sigma
CD11b	550282; BD Biosciences
HNF4α	ab41898; Abcam
CD45	550539; BD Biosciences
CD11cDonkey anti-Rabbit IgG,<br>Alexa Fluor™ 594	# A-21207; Thermo Fisher Scientific
Ly6GDonkey anti-Rat IgG,<br>Alexa Fluor™ 488	# A-21208; Thermo Fisher Scientific
Goat anti-Mouse IgG, Alexa Fluor™ 488	# A-11001; Thermo Fisher Scientific

SUPPLEMENTARY REFERENCES

1. Bangen JM, Hammerich L, Sonntag R, Baues M, Haas U, Lambertz D*, et al.* Targeting CCl4 -induced liver fibrosis by RNA interference-mediated inhibition of cyclin E1 in mice. *Hepatology* 2017, **66**(4)**:** 1242-1257.

2. Bartneck M, Schrammen PL, Mockel D, Govaere O, Liepelt A, Krenkel O*, et al.* The CCR2(+) Macrophage Subset Promotes Pathogenic Angiogenesis for Tumor Vascularization in Fibrotic Livers. *Cell Mol Gastroenterol Hepatol* 2019, **7**(2)**:** 371-390.

3. Movahedi K, Laoui D, Gysemans C, Baeten M, Stange G, Van den Bossche J*, et al.* Different tumor microenvironments contain functionally distinct subsets of macrophages derived from Ly6C(high) monocytes. *Cancer Res* 2010, **70**(14)**:** 5728-5739.

4. Berres ML, Koenen RR, Rueland A, Zaldivar MM, Heinrichs D, Sahin H*, et al.* Antagonism of the chemokine Ccl5 ameliorates experimental liver fibrosis in mice. *J Clin Invest* 2010, **120**(11)**:** 4129-4140.

5. Nevzorova YA, Bangen JM, Hu W, Haas U, Weiskirchen R, Gassler N*, et al.* Cyclin E1 controls proliferation of hepatic stellate cells and is essential for liver fibrogenesis in mice. *Hepatology* 2012, **56**(3)**:** 1140-1149.
